# Liam tackles complex multimodal single-cell data integration challenges

**DOI:** 10.1093/nar/gkae409

**Published:** 2024-06-06

**Authors:** Pia Rautenstrauch, Uwe Ohler

**Affiliations:** Humboldt-Universität zu Berlin, Department of Computer Science, 10099 Berlin, Germany; Max-Delbrück-Center for Molecular Medicine in the Helmholtz Association (MDC), Berlin Institute for Medical Systems Biology (BIMSB), Berlin, Germany; Humboldt-Universität zu Berlin, Department of Computer Science, 10099 Berlin, Germany; Max-Delbrück-Center for Molecular Medicine in the Helmholtz Association (MDC), Berlin Institute for Medical Systems Biology (BIMSB), Berlin, Germany; Humboldt-Universität zu Berlin, Department of Biology, 10099 Berlin, Germany

## Abstract

Multi-omics characterization of single cells holds outstanding potential for profiling the dynamics and relations of gene regulatory states of thousands of cells. How to integrate multimodal data is an open problem, especially when aiming to combine data from multiple sources or conditions containing both biological and technical variation. We introduce liam, a flexible model for the simultaneous horizontal and vertical integration of paired single-cell multimodal data and mosaic integration of paired with unimodal data. Liam learns a joint low-dimensional representation of the measured modalities, which proves beneficial when the information content or quality of the modalities differ. Its integration accounts for complex batch effects using a tunable combination of conditional and adversarial training, which can be optimized using replicate information while retaining selected biological variation. We demonstrate liam’s superior performance on multiple paired multimodal data types, including Multiome and CITE-seq data, and in mosaic integration scenarios. Our detailed benchmarking experiments illustrate the complexities and challenges remaining for integration and the meaningful assessment of its success.

## Graphical Abstract

**Graphical Abstract:**
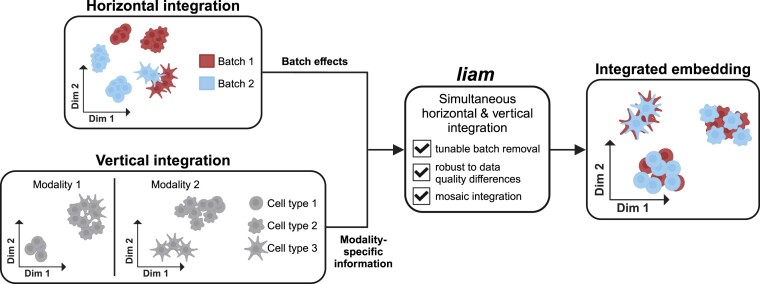

## Introduction

Single-cell omics technologies have transformed how we study cellular systems and are now integral to the biomedical research landscape. Recent advances allow measuring multiple modalities, such as gene expression and chromatin accessibility, from a single cell (1–3). Data from such paired multimodal assays promise unprecedented insight into cellular diversity, the relationships between molecular layers and regulatory processes. However, they also pose complex challenges for data integration. As is the case for unimodal data, intricate study designs and meta-analyses require sophisticated non-linear batch effect removal (horizontal integration, also known as batch effect correction). For paired multimodal data, we additionally need to combine data from distinct modalities that provide complementary information, each with unique dimensionality and statistical properties (vertical integration). Lastly, it is an open challenge to combine paired and unimodal data sets, e.g. from previous studies, into a single representation. Here, we face the additional problem of only partially overlapping cells or features (mosaic integration) (4).

So far, several methods have been developed to solve aspects of these integration problems. For instance, solutions proposed for different unimodal data types (5–8) can be applied to individual modalities of multimodal data. However, they showed varying success on complex horizontal integration tasks in a comprehensive benchmark (5). Likewise, several methods for the vertical integration of multimodal single-cell data are available, but most do not explicitly allow for or have evaluated the success of complex batch effect removal (9–16). If working with more than a single data set, users currently thus have to independently perform horizontal integration of data before applying these tools. A notable exception is the model totalVI, which performs simultaneous horizontal and vertical integration of CITE-seq data using a conditional variational autoencoder (CVAE) (17). Many models for mosaic integration of paired and unimodal data sets (18–22) project the data into a common latent space and explicitly model horizontal integration, but some require additional horizontal integration in the common space (23). Due to their design and underlying assumptions, many of these methods can be expected to be inherently sensitive to large differences in data quality between modalities, a common issue with multimodal data. Related tasks under the umbrella term ‘integration’ include the mapping of multimodal data from a source to a distinct reference modality, e.g. scATAC-seq data to a scRNA-seq reference (‘bridge integration’ (24)), which requires separate batch effect correction for the different modalities and does not take advantage of potential complementary information. Another line of research concerns the imputation of data for non-measured modalities, also called modality translation (25), which can go hand in hand with deriving a shared embedding (19,20,22,23).

As complex integration scenarios become more common, the performance and generalizability of methods are increasingly important. To this end, identifying modeling choices for successful integration of data of varying complexity and quality is critical. Many aspects of multimodal single-cell data integration remain unsolved: it is unclear whether and when projecting data into a common latent space might be more beneficial than separate analyses of different modalities. In addition, managing the trade-off between preserving biological variation and removing undesired batch effects is an open problem, where exploiting meta-information on the experimental design could allow for more principled horizontal data integration.

To tackle these challenges, we develop liam (**l**everaging **i**nformation **a**cross **m**odalities), an adversarial variational autoencoder-based model for paired multimodal single-cell data and mosaic integration, show which modeling choices drive successful integration, and devise new strategies for evaluating integration. To our knowledge, liam is the first approach that allows for a principled tuning of the strength of batch effect removal via combining a conditional VAE with an adversarial training strategy that we recently introduced for the horizontal integration of scATAC-seq data (7), for which we enable scaling. Sample information, such as replicate status or other available meta-information, can guide optimal scaling parameter selection. We apply liam to complex experimental designs with replicate data to confidently assess its integration performance. Liam exhibits state-of-the-art performance, and its early-stage integration strategy leads to superior robustness towards low-quality modalities. We demonstrate its competitive performance on multiple distinct multimodal data sets that include DNA, RNA, and protein measurements. Our contribution exposes and meets the need for models that account for complex real-world study designs with data of varying quality, explores novel avenues for method evaluation, and defines challenges of current benchmarking and evaluation strategies.

## Materials and methods

### Liam: model and software

#### Model description

**Figure 1.**
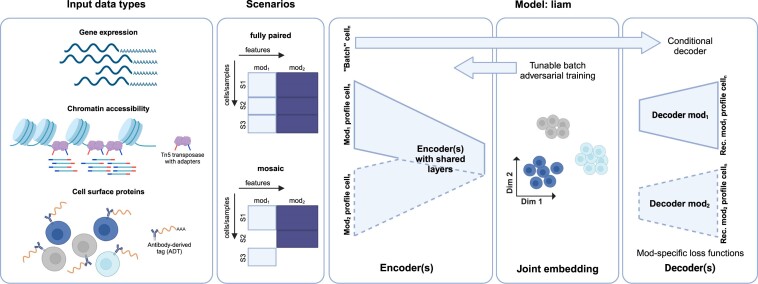
Multimodal single-cell data integration with liam. Liam supports multimodal and unimodal single-cell gene expression, chromatin accessibility, and cell surface protein data. We designed liam for integrating fully paired data sets, or at least one paired with unimodal data sets in a mosaic integration scenario for any number of samples. Given a ‘batch variable’ whose effect to remove from its output, liam performs horizontal (batch) and vertical integration simultaneously. Its output is a batch-corrected joint low-dimensional representation (embedding) of the input modalities. Liam is a variational autoencoder-based model that combines a conditional decoder with a tunable batch adversarial training strategy for horizontal data integration. Other key features are its shared layers in the encoder (‘early-stage integration’), conferring robustness to differences in data quality across integration scenarios, and its modality-specific loss functions. Mod: modality.

VAEs have been successfully employed for the horizontal integration of unimodal data (7,26,27). Here we modify the prototypical VAE framework, building on recent advances in modeling scRNA-seq and scATAC-seq data (7,26,27) and introduce liam (**l**everaging **i**nformation **a**cross **m**odalities), a model for paired multimodal single-cell data integration (horizontal and vertical) and mosaic integration of paired with unimodal data, simultaneously solving these integration tasks (cf. Results section ‘The model liam’, and Figure 1 and Supplementary Figure S1).

##### Encoding

Liam’s encoder has two separate input layers for the two modalities of each cell *n*, followed by one hidden layer each. For cells with a single modality measured (mosaic integration case), we set the input to the non-corresponding network branch to all zero. The output of these hidden layers is fed to separate network branches for modeling cell- and modality-specific size factors, with batch-specific priors (*l*_*n*_ for rna and *d*_*n*_ for atac/adt), which are part of our horizontal data integration strategy. Additionally, we concatenate the output of the two modality-specific hidden layers to model the *k*-dimensional (default: 20) latent variable *z*_*n*_, the low-dimensional cell representation, allowing the model to combine information from both modalities.

##### Decoding

We employ two separate decoders, one per modality. These consist of two hidden layers each, which take a sample from the latent variable *z*_*n*_ and the one-hot encoded batch variable *s*_*n*_ (conditional decoder) as input. In this context, ‘batch’ refers to a meta-information variable, such as the condition or sample of a cell. This way, the model can use the batch information for reconstructing the input data without needing to encode batch-related information in the embedding, which is part of our horizontal data integration strategy. We model gene expression and the CLR-transformed adt counts with a negative binomial distribution, using the implementation of (26) that uses the cell-specific size factor *l*_*n*_. For the chromatin accessibility data, we use the negative multinomial loss, which jointly models a cell’s entire chromatin accessibility profile as introduced in BAVARIA (7), with the modification that we add an extra node to the penultimate fully connected layer, which takes the value of the learned cell-specific atac size factor (*d*_*n*_).

##### Latent factor distributions and inferred parameters

We model cell-specific size factors as log-normally distributed and the latent variable *z*_*n*_ as logistic-normal distributed (17,27), which has the benefit of the latent factors summing to one, allowing for archetype analysis (17,27). Additionally, there are several inferred parameters in the model: the batch-specific per gene/adt dispersion of the negative binomial distribution and the batch-specific dispersion of the negative multinomial loss (batch-specific parameters are part of our horizontal data integration strategy).

##### Adversarial training strategy

To further encourage the model to learn latent representations devoid of batch effects and provide a way of tuning batch-mixing, we employ a batch adversarial training strategy. In particular, we introduce an additional neural network, a batch classifier, as a part of our framework that is trained together with the VAE model. The batch classifier has a single fully-connected hidden layer with 32 nodes that takes as input a sample from *z*_*n*_, which is fed through a gradient reversal layer and predicts the batch from which the sample stems. Using a gradient reversal layer allows us to send a negative feedback to the encoder during joint training when the batch classifier gets better at predicting the batch. We call this default architecture of liam, combining a conditional decoder with the described adversarial training strategy, BAVAE.

##### Further architecture details

All layers are fully-connected layers. We employ dropout layers for the encoder, not for the decoder and batch classifier. We use layer norm and the ReLu activation function for all layers, except for the respective output layers, in which we use other specified nonlinearities (cf. Supplementary Figure S1). A complete schematic representation of the model’s architecture for Multiome data is shown in Supplementary Figure S1. The figure also details all layer dimensions.

##### Difference between Multiome and CITE-seq architecture

For CITE-seq data, the only difference is that the adt-specific encoder has input dimensions equal to the number of adt features, and that the decoder mirrors the rna-specific decoder, with output dimensionality equal to the number of adt features.

#### Loss function fully paired data

The model’s loss function comprises regularization terms for the learned latent factors. In particular, we use the Kullback–Leibler divergence for *z*_*n*_, encouraging *z*_*n*_ to follow a logistic-normal distribution ($z_n \sim \texttt {Logisticnormal}(0,I)$), and for the cell-specific size factors of the distinct modalities *l*_*n*_ and *d*_*n*_, encouraging them to follow a log-normal distribution, using the real mean (*l*_μ_, *d*_μ_) and variance ($l_{\sigma ^2}, d_{\sigma ^2}$) of the log of the mean library size per batch (*s*_*n*_) as priors ($l_n \mid s_n \sim \texttt {Lognormal}(l_{\mu }^\top s_n, l_{\sigma ^2}^\top s_n)$; $d_n \mid s_n \sim \texttt {Lognormal}(d_{\mu }^\top s_n, d_{\sigma ^2}^\top s_n)$). The total regularization loss is:


\begin{equation*} loss_{KL} = KL_{z} + KL_{l} + KL_{d} \end{equation*}

Additionally, the model’s loss function comprises reconstruction loss terms for the distinct modalities. They score the divergence between the input data and the reconstruction with:

1. *loss*_*rna*_ & *loss*_*adt*_≔ Negative binomial loss (26)
2. *loss*_*atac*_ ≔ Negative multinomial loss (7).

Lastly, the loss function comprises an adversarial term that stems from a batch classifier for which we employ a cross-entropy loss between the predicted class (batch) probability and the real batch (*loss*_*adv*_). In some experiments, we use a tunable scaling parameter α, with which we can up-weight the contribution of the loss of the batch classifier in the total loss (α = 1 in liam’s default mode).

We minimize the total loss:

1. For Multiome data: *loss*_*total*_ = *loss*_*rna*_ + *loss*_*atac*_ + *loss*_*KL*_ + α × *loss*_*adv*_
2. For CITE-seq data: *loss*_*total*_ = *loss*_*rna*_ + *loss*_*adt*_ + *loss*_*KL*_ + α × *loss*_*adv*_

If the batch classifier gets better at predicting the correct batch, this gets fed back to the encoder as negative feedback during the backward pass of model training through a gradient reversal layer (28).

#### Loss function mosaic integration

When combining paired and unimodal data (mosaic integration), we set terms with no correspondence in the loss function for cells with only a single modality measured during model training to 0.

#### CVAE variant

For the CVAE variant of liam, we remove the batch classifier network. In addition to feeding the one-hot encoded batch variable to the decoder, we feed it to the encoder layers (except for the bottleneck layer) (conditional encoder).

#### Single-modality model variant

Liam can also be run in a single-modality mode. For this model variant, only the leg of the encoder corresponding to the modality in question is used, and the other is disabled. For the decoder, only the decoder for the respective modality is used.

#### Model training

For training all variants of liam, we chose a mini-batch size of 128 and split the data into a training set comprising 95% of the data and a validation set comprising 5% of the data. We use Adam for optimizing our model parameters, with a learning rate of 1e-3 and weight decay of 1e-6. We employ early stopping with a patience of ten epochs with respect to the validation loss, using the best model for our analyses. We chose 20 dimensions for the latent space for all models, except for the single-modality models used for the concat baseline, for which we used 10. These hyperparameters are set as defaults in the liam package. Models for the competition and mosaic use case were trained using a Tesla-T4 graphic card with CUDA 11.3. Training liam on the competition data set with default parameters took ∼1 h 12 min for random seed 0 (cf. Supplementary note 1.3). Models for the treatment-control and extended treatment-control use case were trained using a Tesla-V100-SXM2-32GB graphic card with CUDA 11.3.

#### Model implementation

Liam is implemented in Python. It employs the scvi-tools library (version 0.14.3) (29), and we used the scvi-tools-skeleton repository (version 0.4.0) as a starting template for package development. It is available as a readily installable open-source Python package with tutorials on GitHub (https://github.com/ohlerlab/liam). The input data to the model needs to adhere to the AnnData format (30).

### Data sets

#### NeurIPS competition data set

The competition use case is based on the phase 2 data of the Multimodal Single-Cell Data Integration NeurIPS competition 2021, accessible through AWS S3. We use this data set to reproduce the competition result, as the preprocessed data available via GEO contains additional data held out during the competition. The data set contains Multiome and CITE-seq data, which comprise gene expression and chromatin accessibility measurements, and gene expression and cell surface protein expression (captured via antibody-derived tags (adt)) measurements, respectively. The data can be retrieved via s3://openproblems-bio/public/phase2-private-data/joint_embedding/openproblems_bmmc_multiome_phase2/ (Multiome) and s3://openproblems-bio/public/phase2-private-data/joint_embedding/openproblems_bmmc_cite_phase2/ (CITE-seq).

The competition organizers purposefully introduced nested batch effects in the study design. This allows for testing the generalization capabilities of computational approaches for horizontal data integration by investigating different levels of batch effects removal, e.g. the removal of inter- versus intra-donor and -site variation. The samples stem from bone marrow mononuclear cells (BMMCs), a complex, disease-relevant, and easily accessible system and 10 distinct donors. The data was generated at four different sites, with samples from one particular donor being measured at four (CITE-seq) and three out of the four (Multiome) sites. For all other donors, a single sample at one site was measured (cf. Supplementary Figure S2C). Each sample is identifiable by a donor site combination (d*s*, with ‘*’ being a wildcard for an identifier for a particular donor and site). Each sample was preprocessed and annotated independently: distinct modalities were preprocessed separately, deriving independent cell type annotations per modality, which were harmonized afterward into one unified annotation per sample. Of note, the cell type annotations are generally marker gene- or cell surface protein marker-based, including the chromatin accessibility modality, for which the organizers derived gene activity matrices from the chromatin accessibility data before marker gene-based annotation (cf. Supplementary note 1.4). A more detailed description of the data set and its preprocessing can be found in appendix A1 of (31).

#### Treatment-control data sets from DOGMA-seq

The treatment-control use case is based on data sets from (32), which are available on GEO (GSE156478). The data sets are multimodal single-cell data sets of peripheral blood mononuclear cells (PBMCs) that were *in vitro* stimulated with anti-CD3/CD28 and a control (unstimulated). We use data from the DOGMA-seq protocol, which measures three modalities at a time, chromatin accessibility, gene expression, and cell surface protein (adt) expression. Two replicates from two different lysis conditions are available (abbreviated as DIG and LLL), which we refer to as Rep1 and Rep2, respectively, resulting in four samples: Rep1_Stim, Rep1_Ctrl, Rep2_Stim and Rep2_Ctrl. For the sole purpose of deriving a feature set for the chromatin accessibility data, we also considered samples from the ASAP-seq technology from the same manuscript, which simultaneously profiles chromatin accessibility and cell surface protein levels, as described below. For more information, see (32).

#### Data sets for the extended treatment-control use case

For the extended treatment-control use case, we collected additional publicly available Multiome data sets of PBMCs. In particular, we use PBMC data from permeabilized cells from (33), available on GEO (GSM5123950) and nuclei from 10x Genomics (https://www.10xgenomics.com/resources/datasets/pbmc-from-a-healthy-donor-granulocytes-removed-through-cell-sorting-10-k-1-standard-1-0-0). We refer to these data sets as Swanson_Multiome_cells and 10x_Multiome_nuclei, respectively. For identifiability, we add the prefix ‘DOGMA’ to the sample names from the treatment-control use case. For the 10x_Multiome_nuclei data set, we obtained expert-derived annotations from ftp://ftp.ebi.ac.uk/pub/databases/mofa/10x_rna_atac_vignette/seurat.rds introduced in https://raw.githack.com/bioFAM/MOFA2_tutorials/master/R_tutorials/10x_scRNA_scATAC.html.

### Data preprocessing

#### Competition data sets

We used the competition data provided as part of the NeurIPS competition. For ADT counts, we used CLR transformed data (across features) (stored in the field adata.X of the provided AnnData object). For gene expression, we used raw counts, and for ATAC, binarized counts (stored in the field adata.layers [‘counts’] in the provided AnnData objects, respectively). The structure of the data set is described in detail in the competition documentation: https://openproblems.bio/neurips_docs/data/dataset/.

#### Derivation of mosaic data set from competition data

We create a mosaic data set from the competition data sets by splitting the data by sequencing site and dropping either all gene expression or all chromatin accessibility features for samples of all but one of the sequencing sites, for which we retain a paired data set. In particular, we drop gene expression features for all samples from sequencing sites 1 and 3 and chromatin accessibility features from samples from sequencing site 2. Samples from site 4 are left unchanged (cf. Figure 6A; corresponds to mosaic scenario: ‘mosaic full’). We split by site to generate an as realistic as possible data set, as we expect technical variation between sites to be larger than between donor variation per site. Additionally, our split retains samples from donor 1 for one of the unimodal chromatin accessibility data sets, the unimodal gene expression data set, and the paired data set, allowing us to more reliably evaluate how well we can integrate these distinct modalities (rna only, atac only and paired).

**Figure 2.**
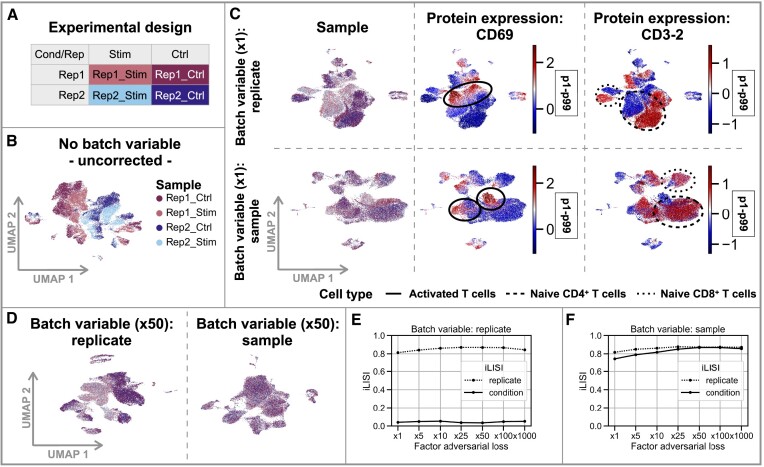
Liam preserves selected treatment effects and improves batch effect removal by exploiting replicates with its adversarial training strategy. Data for all figure panels stems from a treatment-control experiment (T cell stimulation) with replicates (32). (**A**) Experimental design. (**B**) UMAP of an embedding without batch correction. (**C**) UMAPs of embeddings of two models trained with different variables assigned as the batch variable to be removed from the embedding; one uses ‘replicate’ (top row) and the other ‘sample’ (bottom row). Cells are colored by sample and scaled CLR-normalized cell surface protein counts of the T cell activation marker CD69 and another marker predictive of the treatment, CD3-2; different dashed circles highlight cell populations of interest; values outside the p1–p99 percentile range get assigned the min/max value, respectively. (**D**) UMAPs of model variants with increased contribution of the adversarial loss (α: 50) and distinct batch variables colored by sample. (**E, F**) Diversity score iLISI computed for distinct target variables (replicate and condition) for the different variants of liam and increasing contribution of the adversarial loss. The larger the iLISI score, the more mixed samples are for the chosen variable.

**Figure 3.**
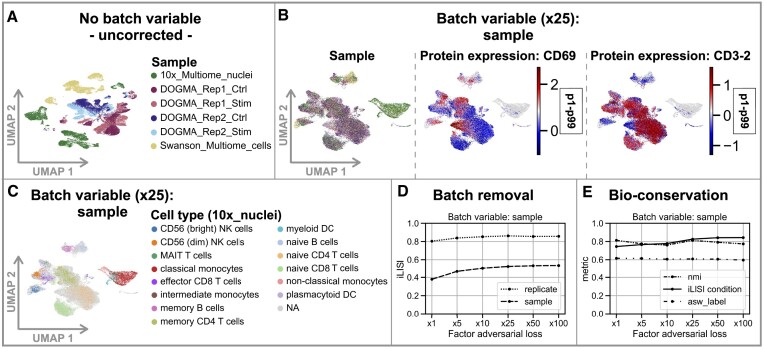
Liam’s batch adversarial training strategy improves batch effect removal while retaining data set-specific variation at the cell type level. Data for all figure panels are PBMC samples from diverse sources (cf. Materials and methods). (**A**) UMAP of an embedding without batch correction. (**B**) UMAPs of embeddings of liam trained with ‘sample’ as batch variable and α: 50. Cells are colored by sample and scaled CLR-normalized cell surface protein counts of the T cell activation marker CD69 and another marker predictive of the treatment, CD3-2; values outside the p1–p99 percentile range get assigned the min/max value, respectively. (**C**) as (B), but cells are colored by expert-derived annotations available for one data set (10x_Multiome_nuclei). (**D, E**) Batch effect removal and bio-conservation performance of model variants with increasing contribution of the adversarial loss across several metrics relying on meta-information available for subsets of the data.

**Figure 4.**
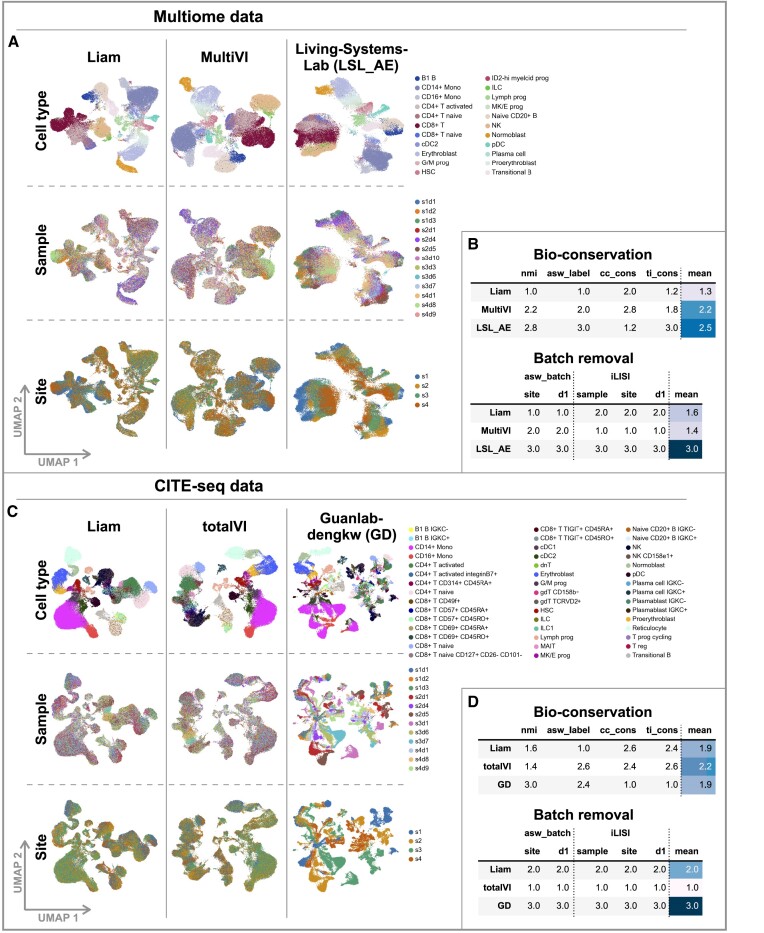
Liam excels in a comprehensive benchmark across distinct data types. Data for all figure panels stems from the NeurIPS 2021 Multimodal Single-Cell Data Integration competition. (**A, C**) UMAPs of embeddings obtained with liam and competitors for (A) Multiome and (C) CITE-seq data; cells are colored by provided cell type annotation (cell type), sample id (sample), and sequencing site (site). (**B, D**) Rank-based model performance on bio-conservation and batch removal metrics for (B) Multiome and (D) CITE-seq data. Each entry corresponds to the mean rank of the model for the respective metric across five random seeds. The absolute metrics scores, including all competition metrics, are shown in Supplementary Figures S7 and S9.

**Figure 5.**
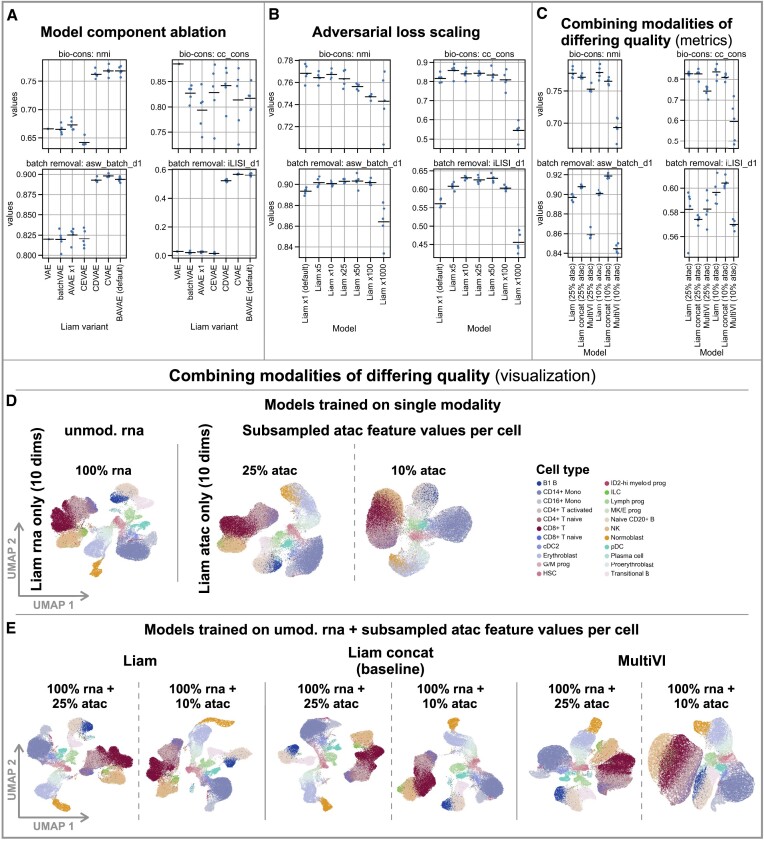
Examining modeling choices exploiting the competition data set. Data for all figure panels stems from the NeurIPS 2021 Multimodal Single-Cell Data Integration competition Multiome data set. (**A**) Evaluation of the influence of modeling choices on batch effect removal through model component ablation via selected performance metrics (bio-conservation: nmi, cc_cons, batch effect removal: asw_batch_d1, iLISI_d1) with the horizontal line indicating the mean. (**B**) Evaluation of the influence of adversarial loss scaling on model performance (Liam: BAVAE). Performance metrics as in (A). (**C–E**) Evaluating model performance when combining modalities of differing quality. (D) and (E) UMAPs of embeddings obtained with distinct models. (D) Left: a variant of liam trained on rna data only, using the entire rna data. Right: variants of liam trained on atac data only using atac subsampled to 25% and 10% of feature values per cell. (E) Models using the unmodified rna data and atac data subsampled to 25% and 10% of feature values per cell, respectively. Liam (default, trained on both modalities), Liam concat (concatenation of embeddings from an rna only (10 dims) and atac only model (10 dims)), and MultiVI. (C) Performance metrics as in (A) for models in (E). All computed metrics, including all competition metrics, are shown in Supplementary Figure S7.

**Figure 6.**
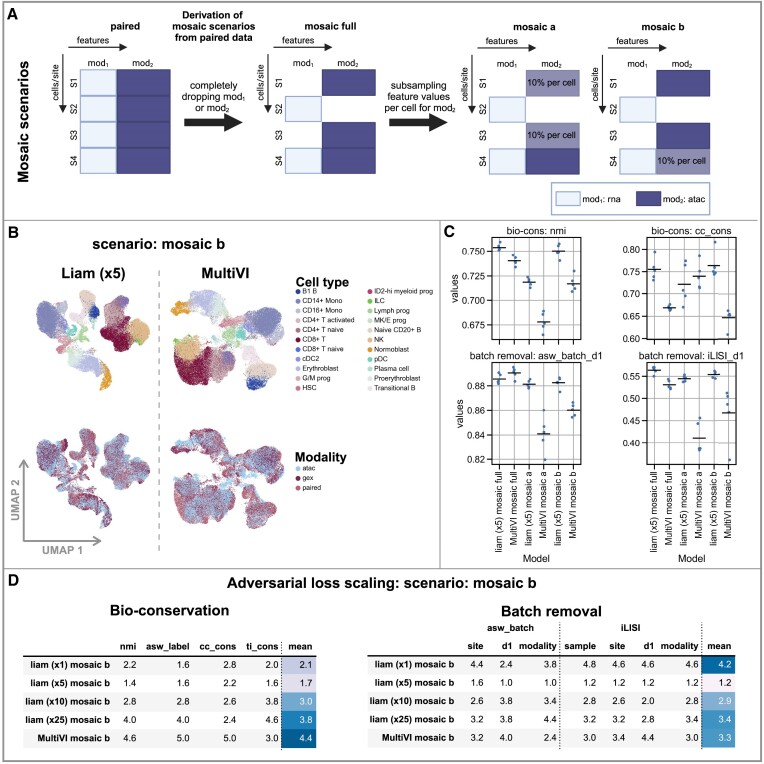
Liam excels at mosaic integration of data with differing quality. We derived data for all figure panels from the NeurIPS 2021 Multimodal Single-Cell Data Integration competition Multiome data set. (**A**) Derivation of the mosaic scenarios from the original data: Dropping entire modalities for samples of some sites (s*) and additionally subsampling the feature values of the atac modality to 10% per cell (cf. Materials and methods). (**B**) UMAPs of embeddings obtained with liam (α: 5) and MultiVI for the scenario ‘mosaic b’ on the entire mosaic data set; cells are colored by provided cell type annotations and modality. (**C**) Selected performance metrics of models for all scenarios (bio-conservation: nmi, cc_cons, batch effect removal: asw_batch_d1, iLISI_d1) with the horizontal line indicating the mean. (**D**) Rank-based performance evaluation of model variants with varying adversarial scaling parameter using bio-conservation and batch removal metrics. Each entry corresponds to the mean rank of the model for the respective metric across five random seeds. All computed metrics, including all competition metrics, are shown in Supplementary Figure S8.

#### Treatment-control data sets from DOGMA-seq

For the treatment-control use case, we reprocessed the author-provided data. Our reprocessing is loosely based on the preprocessing of the original publication (32) but was modified to enable the joint analysis of all DOGMA-seq data sets (samples). Each modality underwent separate quality control, and we retain only cells for which all modalities pass it.

##### Chromatin accessibility

To derive a shared feature set for the chromatin accessibility data from the distinct samples, we jointly analyzed the data from all four DOGMA-seq samples and included chromatin accessibility data from the two ASAP-seq samples from the same study. Starting from the author-provided fragment files, we use an alternative approach to peak calling for feature selection which segments the genome according to cross-cell accessibility profiles called ScregSeg-fi (34). First, we filter each data set independently using ArchR, only retaining cells with a TSS score exceeding four and a minimum of 1000 fragments (ArchR version 1.0.0, R version 4.1.2, reference annotation: hg38 from package BSgenome.Hsapiens.UCSC.hg38 version 1.4.1). Next, we remove cells with high counts exceeding Q3 + 1.5 × IQR for each data set. Afterward, data from all data sets were combined and used for shared feature calling with ScregSeg-fi. We selected 1000 bp bins, only autosomes, and only considered regions with at least one count across all cells and binarized the data. We chose the following parameters for ScregSeg-fi: 7 random runs, HMM with 50 states, and 3000 iterations starting from random initial parameters for each run. As the threshold for informative regions, we chose regions of states that cover at most $1.5\%$ of the genome and that reach a posterior decoding probability of at least 0.9.

##### Gene expression

Starting from author-provided cell-by-feature count matrices, we process each data set separately. First, we remove cells with a total number of unique molecular identifiers (umis) smaller than 1001. After removing low-count cells, we exclude cells with high umi counts. In particular, those that exceed Q3 + 1.5 × IQR. Lastly, we ensure that a minimum of 500 genes was captured per cell and that the percentage of mitochondrial reads is below 30%. As the last step, we exclude mitochondrial genes from the analysis.

##### Cell surface protein expression

Starting from author-provided cell-by-feature count matrices, for each data set, we remove cells that have <101 or >25000 counts, that exceed nine control counts, and that have high CD8 and CD4 expression in the same cell. In particular, a cell cannot have more than 30 CD8 counts and 100 CD4 counts at the same time, considering the antibodies for ‘CD8’ and ‘CD4-1’.

#### Extended treatment-control use case

For the extended treatment-control use case, we reprocessed the additional data to obtain the same feature set across all data sets as input to our model and to mimic the quality control of the original studies, where possible. Each modality underwent separate quality control, and we retain only cells for which all modalities pass it.

##### Chromatin accessibility

For the extended treatment-control use case, we use the same feature set (informative regions) that we previously derived for the treatment-control use case. We create an initial feature count matrix from the provided fragments files with ScregSeg-fi using the scregseg fragments_to_counts function. For the 10x_Multiome_nuclei data, we only keep barcodes called as a cell by the 10x Genomics Cell Ranger ARC pipeline and binarize the data. For the Swanson_Multiome_cells data, we only keep barcodes called as a cell by the 10x Genomics Cell Ranger ARC pipeline and cells that have an ArchR doublet score <1 as reported in the author-provided meta-data file and binarize the data.

##### Gene expression

For the 10x_Multiome_nuclei data, we only retained cells for which a cell type label was available, corresponding to cells that passed the quality control from the original analysis. For the Swanson_Multiome_cells data, we retained cells with >500 and <2750750 feature counts.

### Analyses

For visualizations and analyses, we use scanpy (35), Matplotlib (36) and pandas (37), amongst other Python libraries. Consider the accompanying analysis scripts for a complete list of software dependencies. The graphical abstract and Figures 1, 6A and Supplementary Figure S6A were created with BioRender.com. The leftmost panel of Figure 1 is adapted from ‘ATAC Sequencing’, created by Samara Ona using BioRender.com, and ‘CITE-seq (Cellular Indexing of Transcriptomes and Epitopes by Sequencing) Workflow’, by BioRender.com (2024). Retrieved from https://app.biorender.com/biorender-templates.

#### Treatment-control use case

For the treatment-control use case, we trained several variants of liam, assigning either ‘replicate’ or ‘sample’ as the batch variable. Additionally, we varied the adversarial scaling parameter α (α ∈ {1, 5, 10, 25, 50, 100, 1000}). We trained one model per setting with a random seed of 0.

For visualization purposes, we color cells by CLR transformed, scaled ADT counts not used during model training in Figure 2C, and raw gene expression values in Supplementary Figure S3. We base our broad cell type annotation in Figure 2C on cell surface protein marker expression. Activated T cells: CD69; Naive CD4+ T cells: CD4+, CD27+, CD45RA+, CD45RO−; Naive CD8+ T cells: CD8+, CD27+, CD45RA+, CD45RO−.

#### Extended treatment-control use case

For the extended treatment-control use case, we trained several variants of liam, assigning ‘sample’ as the batch variable and distinct adversarial scaling parameters α (α ∈ {1, 5, 10, 25, 50, 100}). We trained one model per setting with a random seed of 0.

For visualization purposes, we show CLR transformed, scaled ADT counts not used during model training to color cells in Figure 3B.

#### Competition use case

For the competition use case (exception: subsampling analysis, described below), we compare the performance in the joint integration task of liam to variants of liam and several other models. To account for stochasticity in the training processes, we trained five models each, setting a random state component of the respective frameworks to 0, 994, 236, 71 and 415. All corresponding UMAPs show embeddings obtained with a random seed of 0. An exception is the baseline model liam VAE, which was only trained with a random seed of 0.

##### Vertical integration

We compare liam, in which we employ early-stage joint modeling (vertical integration), to simpler baselines. In particular, we compare liam to two single-modality variants of liam with the same dimensionality of the latent space each and a ‘concat’ model. For the concat model, we train two single-modality variants of liam with a latent space dimensionality of half the size of the default model. We concatenate the embeddings obtained from the independently trained single-modality models such that the latent space of the ‘concat’ model space has an equal number of dimensions as liam (default).

- BAVAE rna only (same dimensionality as joint/default; *k* = 20)
- BAVAE atac/adt only (same dimensionality as joint/default; *k* = 20)
- BAVAE concat (concatenation of latent spaces of BAVAE rna + atac/adt only (half dimensionality as joint each; *k* = 10, each)

##### Horizontal integration

To analyze the contribution of individual modeling choices to the model’s horizontal integration capabilities, we systematically ablate individual model components with all models having batch-specific size factors and dispersion parameters (except for VAE). Additionally, we vary the adversarial scaling parameter α. We compare a:

- VAE: no batch correction at all
- batchVAE: VAE + batch-specific cell size factors and dispersion parameters
- CEVAE: conditional encoder only
- CDVAE: conditional decoder only
- CVAE: conditional encoder and decoder
- AVAE x1, x50, x100, x1000*; batch adversarial term only + scaled batch adversarial terms
- BAVAE x1 (default), x5, x10, x25, x50, x100, x1000*; conditional decoder + scaled batch adversarial terms

* numbers indicate scaling parameter α for the adversarial loss

##### Subsampling analysis

Data quality differences pose challenges for data integration. To assess liam’s early-stage vertical integration strategy, we simulate a scenario with substantial quality differences between modalities exploiting the competition data set. For this subsampling analysis, we fix the random seed of the training process to 0 but introduce stochasticity by using five distinct random subsets of the chromatin accessibility data, simulating low-quality data. In particular, we retain 25% of the binarized ATAC feature values per cell, setting the remainder to 0 (random seeds for subsampling: 8831, 234, 11, 9631, 94). We repeat the same procedure retaining 10% of the binarized ATAC feature values per cell. Note that this strategy preserves the overall feature set of the ATAC data; we subsample only individual feature values per cell. The UMAPs in the corresponding figures show embeddings obtained with a random training seed 0 using the random ATAC subsample obtained with seed 8831. This implies that for the concat variant of liam in the subsampling analysis, the 10-dimensional RNA-only data representation is constant (trained with random seed 0), and only the ATAC-only representation has varying input obtained with different random seeds but is trained with a random seed of 0.

##### Comparison with other models

###### MultiVI

We ran MultiVI (22) (scvi-tools version 0.14.3) with default parameters following a tutorial provided by the authors (https://docs.scvi-tools.org/en/stable/tutorials/notebooks/MultiVI_tutorial.html, as of 15 April 2022). Version 0.14.3 was available when we conducted the analysis, and MultiVI was first presented as a preprint (38). The results we obtained with this version of the model should be highly consistent with results one would obtain with the version at the time of MultiVI’s publication (22), as the model itself has not changed (concerning the use case presented), but only its implementation. Of note, this includes a feature preselection step before training the model (also applied in the preprint and final manuscript). In particular, features present in less than 1% of the cells are removed before training. For comparability with liam, we also run a model without feature preselection. The dimensions of the latent space are determined automatically by the model and are dependent on the number of input features. For the model with feature preselection, the latent space has 16 dimensions, for the model without feature preselection, 18 (cf. Supplementary note 1.2).

###### totalVI

We ran totalVI (17) (scvi-tools version 0.14.3) with default parameters following a tutorial provided by the authors (https://docs.scvi-tools.org/en/stable/tutorials/notebooks/totalVI.html, as of 15 April 2022). Of note, this includes total count normalization to 10 000 reads per cell, followed by logarithmization and a feature preselection step before running the model (scanpy version 1.8.2). In particular, for the gene expression modality, the top 4000 highly variable genes were determined with the parameter ‘flavor: seurat_v3’. The latent space dimensionality defaults to 20 dimensions.

###### LSL_AE

Best performing model in original competition framework for task 3 Multiome online category according to competition evaluation criteria (Team name: Living-Systems-Lab; method name: LSL_AE). We adapted the publicly available code from the submissions to the competition (https://github.com/openproblems-bio/neurips2021_multimodal_topmethods/blob/main/src/joint_embedding/methods/lsl_ae/run/script.py) to be compatible with our analyses. The embedding generated by the model has 64 dimensions.

###### Guanlab-dengkw

Best performing model in original competition framework for task 3 CITE-seq online category according to competition evaluation criteria (Team name: Guanlab-dengkw; method name: Guanlab-dengkw (GD)). We adapted the publicly available code from the submissions to the competition https://github.com/openproblems-bio/neurips2021_multimodal_topmethods/blob/main/src/joint_embedding/methods/Guanlab-dengkw/run/script.py to be compatible with our analyses. The embedding generated by the model has 100 dimensions.

###### scVI

scVI (26) is a model for unimodal data integration of scRNA-seq data. We thus apply it to the scRNA-seq modality of the benchmark data set only. We set up two variants of scVI (scvi-tools version 0.14.3). One with default parameters and one with layer norm instead of the default batch norm for the encoder and decoder (cf. Supplementary note 1.1). The latent space dimensionality defaults to 10 dimensions. For training, we selected the same user-defined parameters we used for liam.

#### Mosaic use case

For the scenario ‘mosaic full’, we trained five models each (liam and MultiVI) setting a random state component of the respective frameworks to 8831, 234, 11, 9631 and 94. All corresponding UMAPs show embeddings obtained with a random seed of 11.

##### Subsampling analysis

Further building upon our idea of subsampling the original data to mimic challenging real-world scenarios with poor data quality, we devise two additional scenarios starting from the base scenario ‘mosaic full’. We fix the random seed of the training process to 0 but introduce stochasticity by using five distinct random subsets of the chromatin accessibility data, retaining 10% of the binarized ATAC feature values per cell, setting the remainder to 0 (random seeds for subsampling: 8831, 234, 11, 9631, 94) for either the unimodal chromatin accessibility samples (‘mosaic a’) or the paired modality data sets (‘mosaic b’) (cf. Figure 6A). Note that this strategy preserves the overall feature set of the ATAC data; we subsample only individual feature values per cell, simulating low-quality data. The UMAPs in the corresponding figures show embeddings obtained with a random training seed 0 using the random ATAC subsample obtained with seed 11. Additionally, we investigate whether a joint or separate analysis of paired data with unimodal data of varying quality is more advisable. To this end, we consider different data (sub)sets for the scenarios ‘mosaic a’ and ‘b’. In particular, we train models using the entire mosaic data set (mosaic), unimodal chromatin accessibility data only (atac only), and the paired data and the unimodal gene expression data (rest) (cf. Supplementary Figure S6A).

##### Adversarial loss scaling

We test a range of adversarial scaling parameters α (α ∈ {1, 5, 10, 25}) for the three mosaic scenarios (‘full’, ‘a’, ‘b’) considering the mosaic data (sub)set (cf. Supplementary Figure S6A).

##### Comparison with other models

 

###### MultiVI

C.f. description of MultiVI in section ‘Competition use case’. For the mosaic scenarios, the models’ latent spaces have 15 (‘mosaic full’) and 14 (‘mosaic a’ and ‘mosaic b’) dimensions, we only trained models with feature preselection.

#### Metrics: competition use case

As in the NeurIPS competition, we score batch effect removal (batch removal) and the preservation of biological variation (bio-conservation), to assess model performance. A successful model should rank high for both tasks. We employ the metrics from the NeurIPS competition for bio-conservation but revise its batch removal metrics due to confounding or poor discriminative power (39). For reproducibility, we also report the original batch removal metrics (Supplementary Figures S7-S9) but use less confounded metrics in this study, as explained below. All metrics are implemented in the scib Python package (comprehensively described in (5)) (scib version 1.0.1). We use the full competition metric name here and denote the shorthand used in the manuscript figures and text in parentheses. Overall, we use four metrics to score bio-conservation. Two of them depend on cell type annotations provided by the competition organizers, namely NMI cluster/label (nmi) and cell type ASW (average silhouette width; asw_label). The other two are cell cycle conservation (cc_cons) and trajectory conservation (ti_cons). For batch removal, we report two metrics from the competition for legacy reasons, but disregard them for our performance evaluations: batch ASW (asw_batch_sample), using the sample id as the batch variable, and graph connectivity (graph_conn). We employ batch removal metrics less prone to confounding for performance evaluation. In particular, we use a complementary batch removal metric- iLISI (graph iLISI as implemented in scib v1.0.1 (5)) on the meta-variables site (iLISI_site), and sample id when only considering data from d1 (iLISI_d1). Additionally, we compute the competition metric batch ASW on the variables site (asw_batch_site), and sample id when only considering data from donor 1 (asw_batch_d1). Note that, like the cell type annotation-dependent bio-conservation metrics, the metric batch ASW also requires expert-derived cell type annotations. For donor 1, samples were measured at each site (except for one technical dropout for Multiome data). We compute the batch effect removal metrics only on data from donor 1, as we presume that biological variation between samples from the same donor are minimal. We reckon that subsetting the data to data from the same donor is a good proxy for scoring technical batch effect removal, not penalizing the retention of potential remaining inter-donor variation. For the mosaic integration scenarios, we also compute the metrics iLISI (iLISI_modality) and batch ASW (asw_batch_modality) on the meta-variable modality.

We present absolute metric values in strip plots, where each point represents a model’s performance with a random seed, and horizontal lines denote mean performance across seeds. Additionally, we provide rank-based model comparisons, averaging ranks per metric across random seeds indicative of model consistency. Overall means per model per metric category (bio-conservation and batch removal) highlight relative overall performance.

#### Metrics: treatment-control use case

For quantitatively evaluating horizontal integration success, we computed the iLISI metric (graph iLISI as implemented in scib v1.0.1 (5)) on distinct variables available as meta-information - condition, as a proxy for bio-conservation, and replicate, as a proxy for batch effect removal.

#### Metrics: extended treatment-control use case

For quantitatively evaluating horizontal integration success, we compute several metrics for subsets of the data for which specific meta-information is available. In particular, we compute the iLISI condition and replicate for the treatment-control data, as a proxy for bio-conservation and batch removal. Additionally, we compute the iLISI on the meta-variable sample, available for every data set. Lastly, we compute the clustering metrics nmi and asw_label on cell type annotations available for the 10x data subset, as another proxy for bio-conservation on the cell type level.

## Results

### The model liam

Liam (**l**everaging **i**nformation **a**cross **m**odalities) is a model for the simultaneous horizontal and vertical integration of paired multimodal single-cell data and mosaic integration of paired with unimodal data (Figure 1; Supplementary Figure S1 details architecture). It builds on prior work using variational autoencoders for dimensionality reduction and horizontal integration of unimodal single-cell data (7,26,27) and learns a joint low-dimensional representation (embedding) of two single-cell modalities while accounting for batch effects. Liam currently supports all pairwise combinations of gene expression, chromatin accessibility, and cell surface protein measurements, demonstrated on Multiome and CITE-seq data. We use the negative binomial loss for raw gene expression and CLR-normalized cell surface protein counts and the recently proposed negative multinomial loss for chromatin accessibility data (7).

As we wanted our model to be able to exploit potential correlations and complementarity between modalities, we chose an architecture where the modalities share multiple layers in the encoder that project the data into a joint latent space (‘early-stage integration’; vertical integration). To account for complex batch effects, we model size factors and use a conditional decoder combined with an adversarial training strategy to remove the influence of a specified ‘batch variable’ on the embedding (horizontal integration) (7,28). We introduce a tunable scaling parameter α for the adversarial training strategy, with which we can increase the contribution of the adversarial loss term, allowing us to encourage the mixing of cells with respect to the specified batch variable. By exploiting available meta-information like replicate status, we can choose a parameter that optimizes batch effect removal while preserving biological information.

As in the works of (27) and (17), we employ a logistic-normal distribution for the latent cell variable, making the latent factor loadings interpretable as probabilities. We use layer norm for each layer, as we found that the single modality model scVI (26) achieved better horizontal integration when we chose layer norm instead of batch norm (scVI default) (Supplementary note 1.1). When combining paired and unimodal data (mosaic integration), we set terms with no correspondence in the loss function for cells with only a single modality measured during model training to 0. Note that we also provide variants of liam for unimodal data alone, e.g., gene expression data via scRNA-seq. Liam is available as a readily installable open-source Python package via GitHub, where we provide usage tutorials, and is compatible with the widely-used AnnData data structure (30). Further details on liam’s implementation are provided in the Materials and methods section.

### Preserving selected treatment effects by exploiting replicates

The increasing complexity of study designs requires flexible integration methods that reduce unwanted batch effects while preserving biologically meaningful differences. We designed liam such that it can exploit nested batch effect structure and disentangle technical from biological variation. To illustrate this feature, we apply liam to four data sets from the stimulation of T cells, with two replicates each of treatment and control conditions (32) (Figure 2A and B). It comprises measurements of three modalities from the same cell, chromatin accessibility and gene expression (used for model training), and cell surface proteins (used for model validation). Using meta-information collected during sample preparation, we conduct experiments assigning distinct variables as the ‘batch variable’ in the model (i.e. the variable whose effects to remove from the latent representation), with a batch adversarial training parameter of α set to 1. We can also use this meta-information as a proxy for batch effect removal and biological signal conservation by evaluating how well cells from the different replicates or conditions mix. We assess mixing with the diversity score iLISI (5,40), for which a higher score indicates stronger mixing with respect to the chosen variable.

When assigning the experimental replicate pairs as batch variable, liam removes technical variation between replicates (high iLISI score for variable ‘replicate’) while retaining differences between conditions (i.e. stimulation and control; low iLISI score for variable ‘condition’) (Figure 2E). By contrast, when assigning each sample as a distinct batch, cells from stimulation and control samples within and between replicates are mostly mixed (high iLISI scores for variables ‘replicate’ and ‘condition’), potentially resulting in a loss of biological signal induced by the stimulation (Figure 2F). Both settings preserve known treatment effects, e.g. the emergence of activated T cells marked by the expression of the cell surface protein CD69 after T cell stimulation (Figure 2C). However, more subtle biological differences are only captured by the model penalizing differences between replicates, but not samples. This is reflected in the split of cell populations by condition, coinciding with stimulation-affected cell surface marker expression not used for training (e.g. the selective depletion of CD3-2 cell surface marker expression in stimulated samples) (cf. Figure 2C). In summary, liam can exploit nested batch effect structure to capture more nuanced differences when replicates serve as the batch variable . Nevertheless, even when using individual samples as the batch variable, liam retains cell type-level biological differences, showcasing its applicability for any experimental design.

### Tuning batch adversarial training improves batch effect removal

The presented analyses highlight a common issue of integrating data sets without replicates: When removing batch effects between samples, we face a trade-off between preserving biological variation and removing unwanted batch effects. Assuming that shared variation between multiple replicates profiled under the same condition is more likely to be of biological than of technical origin, we run a low risk of removing biological signal between replicates beyond sampling variation. We thus investigate how tuning the contribution of the adversarial loss compares between choosing replicate or sample as the batch variable. In particular, we test different penalization strengths of batch effects via the scaling parameter α (α ∈ {1, 5, 10, 25, 50, 100, 1000}). Figure 2D–F demonstrates that replicate mixing improves when increasing the contribution of the adversarial component for both model variants (increase of iLISI ‘replicate’). At the same time, liam retains condition-specific (biological) differences across a wide range of scaling parameters when choosing ‘replicate’ as the batch variable (constant value for iLISI ‘condition’ (cf. Figure 2E), and gradually loses them for the model with batch variable ‘sample’ for increasing α (increasing value for iLISI ‘condition’ (cf. Figure 2F). These results demonstrate that liam’s tunable adversarial training effectively preserves biological information and improves batch effect removal across different scaling parameters by utilizing replicate status. If replicates are lacking, other meta-information about the sample, like monitoring the retention of sample-specific cell types (here, stimulated T cells), can guide optimal parameter selection for specific downstream applications.

In typical integration scenarios, batch effects can vary drastically across samples, and replicates are typically unavailable. To assess liam’s performance in such situations, we extended the treatment-control use case to include two additional published PBMC samples with substantial batch effects (10x_Multiome_nuclei, Swanson_Multiome_cells) (cf. Figure 3A). We train liam on this data with ‘sample’ as the batch variable, varying the adversarial scaling parameter α (α ∈ {1, 5, 10, 25, 50, 100}). Besides using the iLISI metric for distinct meta-variables available for (sub)sets of the data as a proxy for batch removal and bio-conservation, we score bio-conservation with the clustering metrics Normalized Mutual Information (nmi) and average silhouette width (asw_label) on cell type annotations provided for the 10x_Multiome_nuclei sample. Despite the absence of replicates, increasing the adversarial loss enhances batch effect removal (as indicated by increased iLISI ‘sample’ and ‘replicate’) (cf. Figure 3D) while preserving biological variation on the cell type level (nmi and asw_label) and gradually diminishing condition-specific differences (iLISI ‘condition’; cf. Figure 3E). At α: 25, with nearly maximal observed batch removal and remaining condition-specific variation, data set-specific cell types are preserved. In particular, we observe distinct clusters for activated T cells for DOGMA_Rep1_Stim and DOGMA_Rep2_Stim, reflected by CD69 protein expression (cf. Figure 3B), and monocytes, depleted in the DOGMA- but present in the 10x_Multiome_nuclei (annotated) and Swanson_Multiome_cells (un-annotated) data sets (cf. Figure 3C). In summary, these analyses demonstrate the benefits of liam’s tunable batch adversarial training strategy across diverse integration scenarios.

### Liam excels in a comprehensive benchmark across distinct data types

To further test liam’s capabilities, we use a benchmark data set from the NeurIPS 2021 competition specifically designed for ‘Multimodal Single-Cell Data Integration’ (31). This data set comprises samples from multiple donors measured at four sites, thus containing within- and across-site and donor-variation (nested batch effects) and reflecting real-world challenges (cf. Supplementary Figure S2C; sample: unique donor-site combination). Its experimental design allows for assessing if methods can handle batch effects of distinct sources and scales. Lastly, the organizers provide expert-derived cell type annotations as a surrogate for ground truth for cellular state, allowing us to evaluate our modeling choices beyond batch effect removal.

Liam participated in the NeurIPS 2021 competition for the task: ‘Jointly learning representations of cellular identity’ (Task 3) and ranked 4th for Multiome and 2nd for CITE-seq data in the online training category. The aim of this task was to learn a low-dimensional data representation from multiple modalities, whose quality was scored with biology conservation and batch removal metrics. Working with the competition organizers, we realized that the competition metrics for evaluating batch effect removal were, unfortunately, either confounded by the nested batch effect structure of the data or had low discriminative power (39). Here, we conduct additional evaluations with batch removal metrics less prone to confounding, complementing the competition’s bio-conservation metrics (cf. Materials and methods). In addition to the models that performed best according to the competition’s evaluation criteria, we considered alternative VAE-based approaches—LSL_AE and MultiVI (22) for Multiome and Guanlab-dengkw (GD) and totalVI (17) for CITE-seq data, respectively. We trained all models using the sample id (composite of site and donor) as the batch variable with no constraints on resource usage.

Figures 4B and D illustrate that liam is highly effective in removing nested batch effects while retaining biological variation for both Multiome and CITE-seq data (absolute scores for all metrics shown in Supplementary Figures S7 and S9). On Multiome data, liam outperforms MultiVI on all and LSL_AE on most bio-conservation metrics (Figure 4B and Supplementary Figure S7). Liam and MultiVI take the lead on one of the two donor 1-specific batch effect removal metrics each (cf. Materials and methods). Both clearly outperform LSL_AE, which fails to remove the nested batch effects between sites (Figure 4B), reflected by the stratification of cells by the site (Figure 4A). The performance advantage over MultiVI is robust to the author-recommended preprocessing choice of feature preselection (Supplementary note 1.2), and liam is around twofold faster than MultiVI (Supplementary note 1.3). On CITE-seq data, liam performs favorably for the annotation-based bio-conservation metrics nmi and asw_label (Figure 4D). For the bio-conservation metrics cc_cons and ti_cons (Figure 4D), GD achieves the best results. Yet, this is rendered irrelevant by the model’s low success in removing nested batch effects and integrating data from different sites, again reflected by the stratification of cells by the site (Figure 4C) and poor performance on the employed batch removal metrics. As for the Multiome data, liam and totalVI outperform the best model from the competition concerning batch integration (GD), with totalVI overall performing slightly better than liam. In summary, liam successfully removes complex batch effects while preserving biological signal for Multiome and CITE-seq data.

All of this comparative benchmarking needs to be considered in the context of a striking observation: a baseline variant of liam, for which we only use RNA as a *single* modality from the Multiome data (cf. Supplementary Figure S2), performs equally well. We address this observation in detail in Supplementary note 1.4. While this observation does not affect the evaluation of horizontal integration and comparison between alternative approaches, it points to the limit of insights that can be gained from benchmarks, especially concerning minor performance differences. Data availability and quality of the individual modalities, the dynamics of the biological system, the granularity and quality of expert annotations, and assumptions behind evaluation metrics may all limit what conclusions can be drawn.

The issue of annotation is also illustrated by liam readily discovering cell types not present in the competition ‘ground truth’. Silhouette scores of individual cells with respect to cell type annotations are typically low in regions in-between cell types. However, we also find a prominent example of a low agreement between the reference annotation and obtained clustering for a group of cells annotated as CD8+ T cells. This group of cells expresses the well-characterized MAIT cell markers KLRB1 and SLC4A10 (41) (Supplementary Figure S3). Combined with the best concordance with the provided cell type annotations, this suggests that the embedding learned by liam captures the cellular states present in the data sets well.

### Examining modeling choices

While the scope of possible evaluations in competitions is limited, the controlled setup of the NeurIPS competition allows us to further dissect modeling choices and gain insights into liam’s strengths. Specifically, we systematically ablate individual model components, with all models except VAE using at least batch-specific cell size factors and dispersion parameters (Materials and methods). The conditional decoder (CDVAE) mainly contributes to the horizontal integration performance, with no clear advantage of adding the adversarial component (BAVAE, default value α: ×1) or a conditional encoder (CVAE), as suggested by the minor performance improvements on the donor 1-specific batch removal metrics (cf. Figure 5A). A conditional encoder VAE (CEVAE) and adversarial VAE (AVAE; α: ×1) alone cannot remove batch effects (Figure 5A and Supplementary Figure S5). We observe comparable performance between scaled variants of the AVAE and our BAVAE (default) model for some bio-conservation and batch removal metrics (α ∈ {50, 100}) (Supplementary Figure S4). Nonetheless, the BAVAE (default) model outperforms all AVAE variants. When assessing the impact of scaling the adversarial contribution in the loss function of liam (BAVAE α ∈ {5, 10, 25, 50, 100, 1000}), we observe considerable improvements of batch effect removal (higher scores for asw_batch_d1 and iLISI_d1), albeit at markedly decreasing performance on the cell type label-dependent metrics nmi and asw_label for α > 25 (cf. Figure 5B and Supplementary Figure S7). We observe excellent bio-conservation at considerably improved batch removal performance across a range of recommendable values α ∈ {5, 10, 25}. This evaluation suggests that liam’s conditional decoder drives horizontal data integration success and can be enhanced by liam’s new tunable adversarial training strategy.

When integrating multimodal samples from distinct sources, data quality can vary considerably between samples and modalities. We reasoned that liam might compensate for technical dropouts by exploiting correlation and complementarity between distinct modalities due to its early-stage (vertical) integration strategy, which contrasts later-stage integration strategies as in MultiVI or in the baseline liam concat. As the evaluation of liam and liam concat led to highly similar results for the high-quality competition Multiome data (Supplementary Figure S5B), we deliberately decreased the information content of the chromatin accessibility modality (atac) by subsampling the atac feature values (peaks) per cell to simulate a scenario where we combine a high-quality with a low-quality modality. In particular, we derive two scenarios, randomly selecting only 25% and 10% of the feature values of the binarized chromatin accessibility data per cell (cf. Materials and methods). This severely diminishes the information content of the chromatin accessibility modality, mimicking low-quality data (cf. Figure 5D). For model training, we provide the unmodified gene expression (rna) and subsampled atac data. In these scenarios, liam performs better on bio-conservation than the concat baseline or MultiVI, with trends reinforcing with diminishing information content (Figure 5C and Supplementary Figure S7). At the same time, we observe a better mixing of batches for all models (asw_batch_d1 and iLISI_d1) with diminishing information content, yet again highlighting the trade-off between bio-conservation and batch effect removal. Importantly, in contrast to MultiVI, liam jointly modeling two modalities performs consistently at least as well as the best performing single modality liam model variant (rna only, 10 or 20 dimensions) (Supplementary Figure S7). This finding suggests that liam’s early-stage integration strategy does not impair the model’s performance when jointly modeling a high- and low-quality modality, which we expect to be critical in real-world settings.

### Liam for mosaic integration

While paired data provides a ground truth on the relationship between distinct modalities, obtaining them remains resource-intensive. Current protocols also frequently require extensive adaptation to new cell types or model systems, especially to ensure sufficient coverage for each of the modalities. Mosaic integration, the integration of samples with only partially overlapping cells or features, is thus not only relevant in light of integrating existing unimodal data sets with paired data but, if successful, also an attractive option for future experimental designs. To evaluate liam’s mosaic integration capabilities, we derive three challenging mosaic scenarios from the NeurIPS competition data. These include two scenarios that revisit our idea of subsampling the original data of one of the modalities per cell. We reciprocally test whether we can integrate a low-quality data modality (subsampled to 10% of atac features values per cell) from a unimodal (‘mosaic a’) or paired assay (‘mosaic b’) with high-quality, unmodified counterparts (cf. Materials and methods and Figure 6A). We compare liam with MultiVI and also address whether jointly or separately analyzing the mosaic data (sub)sets (cf. Supplementary Figure S6A) is more advisable. Based on the batch removal performance across scenarios (Figure 6D and Supplementary Figure S6D and E), we set the liam scaling parameter α to ×5. This is a feasible strategy in real-world scenarios since the required meta-information is commonly available. For the presented scenarios, this strategy results in favorable batch removal and bio-conservation performance.

For mosaic integration, liam consistently outperforms MultiVI on bio-conservation, scoring better on all metrics and scenarios except for cc_cons for the ‘mosaic a’ scenario (Figure 6C and Supplementary Figure S8). Concerning batch effect removal (horizontal integration), MultiVI performs slightly better than liam for ‘mosaic full’ but shows clear deficiency when data quality suffers (Figure 6B and Supplementary Figure S6C), with liam outperforming MultiVI for scenarios ‘mosaic a’ and ‘b’ (Figure 6C). In contrast to liam, which achieves stable horizontal integration performance across both subsampling scenarios, MultiVI’s performance markedly decreases for scenario ‘mosaic a’, where we integrate low-quality single modality data sets (cf. Figure 6C and Supplementary Figure S6C).

Concerning the question of whether a joint or separate analysis of the data (sub)sets is more favorable, liam reaches the highest score for the bio-conservation metric nmi on the paired and rna data subset (rest) for all scenarios (Supplementary Figure S6B). While this reflects that integrating the chromatin accessibility data leads to worse preservation of the reference annotations in all scenarios, we believe that integrating high-quality unimodal chromatin accessibility in the ‘mosaic b’ scenario is beneficial for downstream analyses such as transcription factor motif analysis (cf. Supplementary note 1.4, where we discuss issues with gene-expression centric annotation strategies). In both mosaic scenarios, we gain by integrating unimodal with paired data (mosaic (sub)set) compared to analyzing the unimodal chromatin accessibility data alone (atac only, Supplementary Figure S6B). Overall, liam shows excellent performance on mosaic integration, and its early-stage integration renders it more robust to differences in data quality than later-stage integration as in MultiVI.

## Discussion

Liam is a highly flexible model for the simultaneous horizontal and vertical integration of single-cell multimodal data, including mosaic integration of paired multimodal and unimodal data. In contrast to many established models for paired multimodal single-cell data, which require an independent horizontal integration of the distinct modalities before vertical integration (9–11), liam and other VAE-based approaches solve both tasks at once.

Available multi-omics (benchmark) profiling is strongly biased towards well-studied cell types in human, such as peripheral blood mononuclear cells (PBMCs), for which high-quality data has been obtained on different platforms. Recent work on a wider range of cell types and species shows that generating similar-quality data proves difficult. Simulating low-quality data allowed us to distinguish architectural choices. We find that liam’s early-stage integration strategy confers robustness to differences in data quality, with liam outperforming current alternatives on both the paired and mosaic integration scenario. Liam is thus a much-needed complementary approach.

A problem for current multimodal method development and evaluation remains the considerable uncertainty associated with annotations of cellular identity used as a surrogate for ground truth. Since they are usually expert-derived from the data itself, they are subject to available knowledge and varying data quality and will, as such, be biased towards specific preprocessing strategies and better-studied modalities. For the NeurIPS competition data set, which we chose to illustrate liam’s capabilities, the organizers aimed to minimize biasing choices concerning integration approaches and modality. Regardless, the data set remains vulnerable to the problems mentioned, highlighted by the unannotated MAIT cell population in the Multiome data set and the *on par* performance of an RNA-only model with the joint model on the Multiome data (see Supplementary note 1.4). To combat this problem, we suggest scrutinizing and updating benchmarks with new insights (e.g., our use of revised batch removal metrics), considering multiple independent use cases, and possibly limiting future evaluations to cells that can be reliably annotated across several preprocessing strategies. In general, cell type annotation may not be the most insightful benchmark task for multimodal methods, where the impact of successful integration may be better discernible in downstream tasks such as trajectory inference or network reconstruction (42). However, ground truth annotations for those are even harder to obtain.

Increasingly complex study designs require flexible horizontal data integration strategies. Liam can generally account for complex batch effects, and its tunable adversarial training strategy allows for further optimizing batch effect removal, leveraging available meta-information. While acknowledging limitations related to the availability of meta-information and the trade-off of bio-conservation vs. batch effect removal, we provide a first-of-its-kind tunable solution, and our extended experiments support the effectiveness, robustness, and adaptability of liam’s batch adversarial training strategy across diverse use cases. Our experiments suggest that upscaling the adversarial loss is beneficial in various scenarios, improving batch effect removal and demonstrably retaining biological variation on the cell type level across wide parameter ranges. Notably, even without batch adversarial scaling, liam consistently outperforms alternative models in most of our evaluations.

Generative models, like liam, inherently lend themselves to imputing missing data (17,20,22). For instance, (25) treat data imputation as a translation problem and develop an interoperable model trained on paired data. Other methods predict multimodal measurements by averaging across neighboring cells in the joint embedding with the respective modality measured (19,23). This approach can, in principle, be applied to any method deriving a common embedding. However, the value of data imputation is arguably the largest once it generalizes to unmeasured cell types or conditions, which is still an open problem even for single modalities. Undoubtedly, additional simultaneously measured modalities offer new possibilities for downstream analyses, and some approaches provide trimodal data integration (20–23). Whether deriving a common embedding is advisable has not been systematically investigated. Lastly, the rapid developments in the field of natural language processing have found their way into genomics. First studies hint at the potential of transformer-based models for single-cell data integration (43,44). We believe that standardized benchmarking is critical to making the most of these exciting advances and enabling their application in practice.

In summary, liam provides a robust, flexible, and extendable framework for multimodal data integration. Its early-stage integration and tunable batch adversarial training strategy provide demonstrable competitive strengths, making it a method of choice for multimodal single-cell data integration, especially when data quality is uneven among samples or modalities. Further, our analyses highlight significant issues for benchmarking multimodal data integration and provide initial suggestions for meaningful evaluation.

## Supplementary Material

gkae409_Supplemental_File

## Data Availability

The liam software is freely available under a BSD-3-Clause License at https://github.com/ohlerlab/liam. For reference, we make scripts and notebooks for data preprocessing, analyses, and figures available at: https://doi.org/10.5281/zenodo.11084186. Legacy software used for the analyses is available at: https://github.com/ohlerlab/liam_challenge_reproducibility and https://doi.org/10.5281/zenodo.11085436. All data used in this manuscript is publicly available. The data for the competition use case is available via s3://openproblems-bio/public/phase2-private-data/joint embedding/. The data for the treatment-control use case is available via GEO (GSE156478). The data sets for the extended treatment-control use case are available via GEO (GSM5123950), and https://www.10xgenomics.com/resources/datasets/pbmc-from-a-healthy-donor-granulocytes-removed-through-cell-sorting-10-k-1-standard-1-0-0. For more details, refer to Materials and methods.

## References

[1] Ma A. , McDermaid A., Xu J., Chang Y., Ma Q. Integrative methods and practical challenges for single-cell multi-omics. Trends Biotechnol. 2020; 38:1007–1022. 32818441 10.1016/j.tibtech.2020.02.013 PMC7442857

[2] Lee J. , Hyeon D.Y., Hwang D. Single-cell multiomics: technologies and data analysis methods. Exp. Mol. Med. 2020; 52:1428–1442. 32929225 10.1038/s12276-020-0420-2 PMC8080692

[3] Peng A. , Mao X., Zhong J., Fan S., Hu Y. Single-cell multi-omics and its prospective application in cancer biology. Proteomics. 2020; 20:1900271. 10.1002/pmic.201900271 32223079

[4] Argelaguet R. , Cuomo A.S.E., Stegle O., Marioni J.C. Computational principles and challenges in single-cell data integration. Nat. Biotechnol. 2021; 39:1202–1215. 33941931 10.1038/s41587-021-00895-7

[5] Luecken M.D. , Büttner M., Chaichoompu K., Danese A., Interlandi M., Mueller M.F., Strobl D.C., Zappia L., Dugas M., Colomé-Tatché M. et al. Benchmarking atlas-level data integration in single-cell genomics. Nat. Methods. 2022; 19:41–50. 34949812 10.1038/s41592-021-01336-8 PMC8748196

[6] Cao Y. , Fu L., Wu J., Peng Q., Nie Q., Zhang J., Xie X. SAILER: scalable and accurate invariant representation learning for single-cell ATAC-seq processing and integration. Bioinformatics. 2021; 37:i317–i326. 34252968 10.1093/bioinformatics/btab303 PMC8275346

[7] Kopp W. , Akalin A., Ohler U. Simultaneous dimensionality reduction and integration for single-cell ATAC-seq data using deep learning. Nat. Mach. Intell. 2022; 4:162–168.

[8] Ashuach T. , Reidenbach D.A., Gayoso A., Yosef N. PeakVI: a deep generative model for single-cell chromatin accessibility analysis. Cell Rep. Methods. 2022; 2:100182. 35475224 10.1016/j.crmeth.2022.100182 PMC9017241

[9] Argelaguet R. , Arnol D., Bredikhin D., Deloro Y., Velten B., Marioni J.C., Stegle O. MOFA+: a statistical framework for comprehensive integration of multi-modal single-cell data. Genome Biol. 2020; 21:111. 32393329 10.1186/s13059-020-02015-1 PMC7212577

[10] Singh R. , Hie B.L., Narayan A., Berger B. Schema: metric learning enables interpretable synthesis of heterogeneous single-cell modalities. Genome Biol. 2021; 22:131. 33941239 10.1186/s13059-021-02313-2 PMC8091541

[11] Hao Y. , Hao S., Andersen-Nissen E., Mauck W.M., Zheng S., Butler A., Lee M.J., Wilk A.J., Darby C., Zager M. et al. Integrated analysis of multimodal single-cell data. Cell. 2021; 184:3573–3587. 34062119 10.1016/j.cell.2021.04.048 PMC8238499

[12] Zuo C. , Chen L. Deep-joint-learning analysis model of single cell transcriptome and open chromatin accessibility data. Briefings Bioinf. 2021; 22:bbaa287. 10.1093/bib/bbaa287 PMC8293818 33200787

[13] Minoura K. , Abe K., Nam H., Nishikawa H., Shimamura T. A mixture-of-experts deep generative model for integrated analysis of single-cell multiomics data. Cell Rep. Methods. 2021; 1:100071. 35474667 10.1016/j.crmeth.2021.100071 PMC9017195

[14] Cheng M. , Li Z., Costa I.G. MOJITOO: a fast and universal method for integration of multimodal single-cell data. Bioinformatics. 2022; 38:i282–i289. 35758807 10.1093/bioinformatics/btac220 PMC9235504

[15] Duren Z. , Chang F., Naqing F., Xin J., Liu Q., Wong W.H. Regulatory analysis of single cell multiome gene expression and chromatin accessibility data with scREG. Genome Biol. 2022; 23:114. 35578363 10.1186/s13059-022-02682-2 PMC9109353

[16] Li G. , Fu S., Wang S., Zhu C., Duan B., Tang C., Chen X., Chuai G., Wang P., Liu Q. A deep generative model for multi-view profiling of single-cell RNA-seq and ATAC-seq data. Genome Biol. 2022; 23:20. 35022082 10.1186/s13059-021-02595-6 PMC8756637

[17] Gayoso A. , Steier Z., Lopez R., Regier J., Nazor K.L., Streets A., Yosef N. Joint probabilistic modeling of single-cell multi-omic data with totalVI. Nat. Methods. 2021; 18:272–282. 33589839 10.1038/s41592-020-01050-x PMC7954949

[18] Gong B. , Zhou Y., Purdom E. Cobolt: integrative analysis of multimodal single-cell sequencing data. Genome Biol. 2021; 22:351. 34963480 10.1186/s13059-021-02556-z PMC8715620

[19] Kriebel A.R. , Welch J.D. UINMF performs mosaic integration of single-cell multi-omic datasets using nonnegative matrix factorization. Nat. Commun. 2022; 13:780. 35140223 10.1038/s41467-022-28431-4 PMC8828882

[20] Du J.-H. , Cai Z., Roeder K. Robust probabilistic modeling for single-cell multimodal mosaic integration and imputation via scVAEIT. Proc. Natl. Acad. Sci. U.S.A. 2022; 119:e2214414119. 36459654 10.1073/pnas.2214414119 PMC9894175

[21] Zhang Z. , Sun H., Mariappan R., Chen X., Chen X., Jain M.S., Efremova M., Teichmann S.A., Rajan V., Zhang X. scMoMaT jointly performs single cell mosaic integration and multi-modal bio-marker detection. Nat. Commun. 2023; 14:384. 36693837 10.1038/s41467-023-36066-2 PMC9873790

[22] Ashuach T. , Gabitto M.I., Koodli R.V., Saldi G.-A., Jordan M.I., Yosef N. MultiVI: deep generative model for the integration of multimodal data. Nat. Methods. 2023; 20:1222–1231. 37386189 10.1038/s41592-023-01909-9 PMC10406609

[23] Ghazanfar S. , Guibentif C., Marioni J.C. Stabilized mosaic single-cell data integration using unshared features. Nat. Biotechnol. 2024; 42:284–292. 37231260 10.1038/s41587-023-01766-z PMC10869270

[24] Hao Y. , Stuart T., Kowalski M.H., Choudhary S., Hoffman P., Hartman A., Srivastava A., Molla G., Madad S., Fernandez-Granda C. et al. Dictionary learning for integrative, multimodal and scalable single-cell analysis. Nat. Biotechnol. 2024; 42:293–304. 37231261 10.1038/s41587-023-01767-y PMC10928517

[25] Wu K.E. , Yost K.E., Chang H.Y., Zou J. BABEL enables cross-modality translation between multiomic profiles at single-cell resolution. Proc. Natl. Acad. Sci. U.S.A. 2021; 118:e2023070118. 33827925 10.1073/pnas.2023070118 PMC8054007

[26] Lopez R. , Regier J., Cole M.B., Jordan M.I., Yosef N. Deep generative modeling for single-cell transcriptomics. Nat. Methods. 2018; 15:1053–1058. 30504886 10.1038/s41592-018-0229-2 PMC6289068

[27] Svensson V. , Gayoso A., Yosef N., Pachter L. Interpretable factor models of single-cell RNA-seq via variational autoencoders. Bioinformatics. 2020; 36:3418–3421. 32176273 10.1093/bioinformatics/btaa169 PMC7267837

[28] Ganin Y. , Ustinova E., Ajakan H., Germain P., Larochelle H., Laviolette F., Marchand M., Lempitsky V. Domain-adversarial training of neural networks. J. Mach. Learn. Res. 2016; 17:35.

[29] Gayoso A. , Lopez R., Xing G., Boyeau P., Valiollah Pour Amiri V., Hong J., Wu K., Jayasuriya M., Mehlman E., Langevin M. et al. A Python library for probabilistic analysis of single-cell omics data. Nat. Biotechnol. 2022; 40:163–166. 35132262 10.1038/s41587-021-01206-w

[30] Virshup I. , Rybakov S., Theis F.J., Angerer P., Wolf F.A. anndata: annotated data. 2021; bioRxiv doi:19 December 2021, preprint: not peer reviewed 10.1101/2021.12.16.473007.

[31] Luecken M.D. , Burkhardt D.B., Cannoodt R., Lance C., Agrawal A., Aliee H., Chen A.T., Deconinck L., Detweiler A.M., Granados A. et al. A sandbox for prediction and integration of DNA, RNA, and proteins in single cells. Thirty-fifth Conference on Neural Information Processing Systems: Datasets and Benchmarks Track (Round 2). 2021;

[32] Mimitou E.P. , Lareau C.A., Chen K.Y., Zorzetto-Fernandes A.L., Hao Y., Takeshima Y., Luo W., Huang T.-S., Yeung B.Z., Papalexi E. et al. Scalable, multimodal profiling of chromatin accessibility, gene expression and protein levels in single cells. Nat. Biotechnol. 2021; 39:1246–1258. 34083792 10.1038/s41587-021-00927-2 PMC8763625

[33] Swanson E. , Lord C., Reading J., Heubeck A.T., Genge P.C., Thomson Z., Weiss M.D., Li X.-j., Savage A.K., Green R.R. et al. Simultaneous trimodal single-cell measurement of transcripts, epitopes, and chromatin accessibility using TEA-seq. eLife. 2021; 10:e63632. 33835024 10.7554/eLife.63632 PMC8034981

[34] McGarvey A.C. , Kopp W., Vučićević D., Mattonet K., Kempfer R., Hirsekorn A., Bilić I., Gil M., Trinks A., Merks A.M. et al. Single-cell-resolved dynamics of chromatin architecture delineate cell and regulatory states in zebrafish embryos. Cell Genomics. 2022; 2:100083. 36777038 10.1016/j.xgen.2021.100083 PMC9903790

[35] Wolf F.A. , Angerer P., Theis F.J. SCANPY: large-scale single-cell gene expression data analysis. Genome Biol. 2018; 19:15. 29409532 10.1186/s13059-017-1382-0 PMC5802054

[36] Hunter J.D. Matplotlib: a 2D graphics environment. Comput. Sci. Eng. 2007; 9:90–95.

[37] McKinney W. van der Walt S. , Millman J. Data structures for statistical computing in Python. Proceedings of the 9th Python in Science Conference. 2010; 56–61.

[38] Ashuach T. , Gabitto M.I., Jordan M.I., Yosef N. MultiVI: deep generative model for the integration of multi-modal data. 2021; bioRxiv doi:20 August 2021, preprint: not peer reviewed 10.1101/2021.08.20.457057. PMC10406609 37386189

[39] Lance C. , Luecken M.D., Burkhardt D.B., Cannoodt R., Rautenstrauch P., Laddach A., Ubingazhibov A., Cao Z.-J., Deng K., Khan S. et al. Kiela D. , Ciccone M., Caputo B. Multimodal single cell data integration challenge: results and lessons learned. Proceedings of the NeurIPS 2021 Competitions and Demonstrations Track, Vol. 176 of Proceedings of Machine Learning Research. 2022; 162–176.

[40] Korsunsky I. , Millard N., Fan J., Slowikowski K., Zhang F., Wei K., Baglaenko Y., Brenner M., Loh P.-R., Raychaudhuri S. Fast, sensitive and accurate integration of single-cell data with Harmony. Nat. Methods. 2019; 16:1289–1296. 31740819 10.1038/s41592-019-0619-0 PMC6884693

[41] Park D. , Kim H.G., Kim M., Park T., Ha H.-H., Lee D.H., Park K.-S., Park S.J., Lim H.J., Lee C.H. Differences in the molecular signatures of mucosal-associated invariant T cells and conventional T cells. Sci. Rep. 2019; 9:7094. 31068647 10.1038/s41598-019-43578-9 PMC6506535

[42] Li C. , Virgilio M.C., Collins K.L., Welch J.D. Multi-omic single-cell velocity models epigenome–transcriptome interactions and improves cell fate prediction. Nat. Biotechnol. 2023; 41:387–398. 36229609 10.1038/s41587-022-01476-y PMC10246490

[43] Cui H. , Wang C., Maan H., Duan N., Wang B. scFormer: a universal representation learning approach for single-cell data using transformers. 2022; bioRxiv doi:22 November 2022, preprint: not peer reviewed 10.1101/2022.11.20.517285.

[44] Cui H. , Wang C., Maan H., Pang K., Luo F., Wang B. scGPT: towards building a foundation model for single-cell multi-omics using generative AI. 2023; bioRxiv doi:01 May 2023, preprint: not peer reviewed 10.1101/2023.04.30.538439. 38409223

