# Supplementary material for: Liam tackles complex multimodal single-cell data integration challenges
Source: Nucleic Acids Res. 2024 Jun 6;52(12):e52. doi: 10.1093/nar/gkae409 (PMC11229356; doi:10.1093/nar/gkae409)
Supplement: gkae409_Supplemental_File [file gkae409_supplemental_file.pdf]

Supplementary notes and figures to Liam tackles complex multimodal single-cell data integration challenges

Pia Rautenstrauch[1,2] and Uwe Ohler[1,2,3,*]

[1]Humboldt-Universität zu Berlin, Department of Computer Science, 10099 Berlin, Germany
[2]Max-Delbrück-Center for Molecular Medicine in the Helmholtz Association (MDC), Berlin Institute for Medical Systems Biology (BIMSB), Berlin, Germany
[3]Humboldt-Universität zu Berlin, Department of Biology, 10099 Berlin, Germany
[*]Corresponding author and lead contact, uwe.ohler@mdc-berlin.de

1 Supplementary notes

1.1 Layer normalization leads to favorable performance over batch normalization

During the initial stages of model development, we tested different normalization layers within the scVI framework [1], an RNA-only model, using the treatment-control dataset (utilizing only the RNA modality; results not shown). Our analyses indicated that layer normalization outperformed batch normalization (the default in scVI) in terms of horizontal integration performance. We thus used layer normalization in our model, liam. Post hoc, we confirmed this observation using the competition dataset, which includes cell type labels, enabling a comprehensive performance evaluation. We compared scVI with its default settings (using batch normalization) to a variant using layer normalization, observing a significant improvement in batch effect removal and bio-conservation across various metrics, except for cell cycle conservation (cc_cons) (Figure S7).

1.2 Effect of feature preselection on MultiVI's performance

In our analyses involving MultiVI, we followed the authors' recommendations entailing a feature preselection step, recommending to use of only features present in more than 1% of the cells. We wanted to rule out that this feature preselection step is a major contributing factor to the observed differences in model performance. Hence, we trained a MultiVI model without feature preselection (abbreviated as 'no fp' in figures) but otherwise identical settings on the competition use case data. Note that the number of dimensions of the resulting embedding differs, as MultiVI automatically determines it from the number of input features. Figure S7 shows how omitting feature preselection affects the performance metrics. All trends observed with MultiVI with feature preselection are preserved for MultiVI without feature preselection. Liam outperforms both MultiVI variants on bio-conservation and does better on the average silhouette width (asw)-based batch removal metrics. The MultiVI variants do better on the iLISI-based batch removal metrics.

1.3 Comparing convergence and runtime of liam and MultiVI

While MultiVI [2] models chromatin accessibility data with a Bernoulli distribution, we use a negative multinomial distribution for liam. We observe that MultiVI needed substantially more epochs to converge on the competition use case data. For liam, the average number of epochs until early stopping was: $54.8 \pm 4.09\sigma$, for MultiVI with default settings: $212.4 \pm 27.11\sigma$, and for MultiVI no feature preselection: $189.8 \pm 9.47\sigma$. For random seed 0, liam converged in 50 epochs and trained for $\sim$ 1h 12min. MultiVI converged in 220 epochs and trained for $\sim$ 2h 28mins.

1.4 Limitations of current benchmarks concerning vertical integration evaluation

As part of our method evaluation, we compared the performance of liam jointly modeling both modalities of the paired NeurIPS competition data sets (liam default) to variants of liam that use only one of the individual modalities each (baseline). Unfortunately, this baseline crucial for understanding a method's advantages is rarely computed. Figure S2 shows that the RNA-only model performs on a par with, if not slightly better than the joint model with which we participated in the competition for the Multiome data. Given that the competition was set up to score multimodal data integration, it may appear discouraging that a model trained on a single modality seemed to perform best in the framework. The small performance difference between the joint and the RNA-only model highlights an important limitation in the expressiveness of the benchmark for evaluating vertical integration: While it is possible that the RNA-only model indeed performs best, another possibility is that the RNA-only model's superior performance is an artifact of our (RNA) gene expression-centric prior knowledge affecting the definition of cellular states. The competition organizers derived the cell type labels used for evaluation (bio-consevation: nmi and asw_label; batch removal: asw_batch) independently per data set and modality and harmonized them afterward. With this strategy, Luecken et al. [3] attempted to capture data set-specific substructure in the final cell type annotations. Regardless, the annotations for the Multiome data set are gene expression-centric, as the chromatin accessibility

data was converted to gene activity (GA) scores, thus largely ignoring information from intergenic cell type-specific regulatory elements, and the subsequent annotation is based on known GEX markers. In fact, it has been shown that reducing chromatin accessibility signal to GA matrices before dimensionality reduction results in a substantial information loss (discussed in [4]).

The on par performance of an RNA-only model with the joint model on the Multiome data on cell type label-dependent bio-conservation metrics also suggests that we cannot recover more information on the provided cell type level with a joint than with an RNA-only model for the Multiome data set. For the Multiome data, any existing substructure that a joint model might better recover seems to be less than the overall uncertainty and noise in the cell type labels, leading to all models hitting a maximal performance at around 0.6 for the metric asw_label. Pre-trained models seem to have been able to achieve slightly higher scores [5], but the incompleteness of the annotations mentioned earlier (e.g., the missed MAIT cell population (Figure S3)) let us question the meaningfulness of these results. Additionally, we currently still lack expressive metrics capturing modality-specific information, as the GEX-based cc_cons metric [4], on which the ATAC-only model performed notably worse. This is in line with Ma et al. [6], who observed a less localized cell cycle signature in an embedding derived from scATAC-seq data compared to an embedding derived from scRNA-seq data when treating modalities from paired data from the same cell independently.

The observed limitations are slightly alleviated for CITE-seq data, where the joint model performed better on capturing the harmonized reference cell type annotations. This is in line with previous observations of some populations being better discernible with ADT than GEX and vice versa but may be different for other biological systems with less well-defined cell surface markers and antibody availability.

We tried to circumvent these limitations by exploring alternative ways to use the benchmark data, simulating challenging (real-world) conditions of differing coverage (data quality) across modalities to test different modeling choices. Our results suggest that, in practice, joint modeling may be beneficial. In any case, all our model variants do well on horizontal integration and seem to capture the overall biological information contained in the data, especially the RNA-only model for Multiome data, and the joint models. It will be interesting to use the embeddings derived with liam as a starting point to explore one of the main benefits of the paired data, ground truth on relationships between modalities.

References

[1] Lopez R, Regier J, Cole MB, Jordan MI, Yosef N. Deep generative modeling for single-cell transcriptomics. Nat Methods. 2018;15(12):1053-8. Available from: https://doi.org/10.1038/s41592-018-0229-2.

[2] Ashuach T, Gabitto MI, Koodli RV, Saldi GA, Jordan MI, Yosef N. MultiVI: deep generative model for the integration of multimodal data. Nat Methods. 2023;20(8):1222-31. Available from: https://doi.org/10.1038/s41592-023-01909-9.

[3] Luecken MD, Burkhardt DB, Cannoodt R, Lance C, Agrawal A, Aliee H, et al. A sandbox for prediction and integration of DNA, RNA, and proteins in single cells. In: Thirty-fifth Conference on Neural Information Processing Systems Datasets and Benchmarks Track (Round 2); 2021. Available from: https://openreview.net/forum?id=gN35BGa1Rt.

[4] Rautenstrauch P, Vlot AHC, Saran S, Ohler U. Intricacies of single-cell multi-omics data integration. Trends Genet. 2022;38(2):128-39. Available from: https://doi.org/10.1016/j.tig.2021.08.012.

[5] Lance C, Luecken MD, Burkhardt DB, Cannoodt R, Rautenstrauch P, Laddach A, et al. Multimodal single cell data integration challenge: Results and lessons learned. In: Kiela D, Ciccone M, Caputo B, editors. Proceedings of the NeurIPS 2021 Competitions and Demonstrations Track. vol. 176 of Proceedings of Machine Learning Research. PMLR; 2022. p. 162-76. Available from: https://proceedings.mlr.press/v176/lance22a.html.

[6] Ma S, Zhang B, LaFave LM, Earl AS, Chiang Z, Hu Y, et al. Chromatin Potential Identified by Shared Single-Cell Profiling of RNA and Chromatin. Cell. 2020;183(4):1103-16.e20. Available from: https://doi.org/10.1016/j.cell.2020.09.056.

[7] Gayoso A, Steier Z, Lopez R, Regier J, Nazor KL, Streets A, et al. Joint probabilistic modeling of single-cell multi-omic data with totalVI. Nat Methods. 2021;18(3):272-82. Available from: https://doi.org/10.1038/s41592-020-01050-x.

[8] Park D, Kim HG, Kim M, Park T, Ha HH, Lee DH, et al. Differences in the molecular signatures of mucosal-associated invariant T cells and conventional T cells. Sci Rep. 2019;9(1):7094. Available from: http://www.nature.com/articles/s41598-019-43578-9.

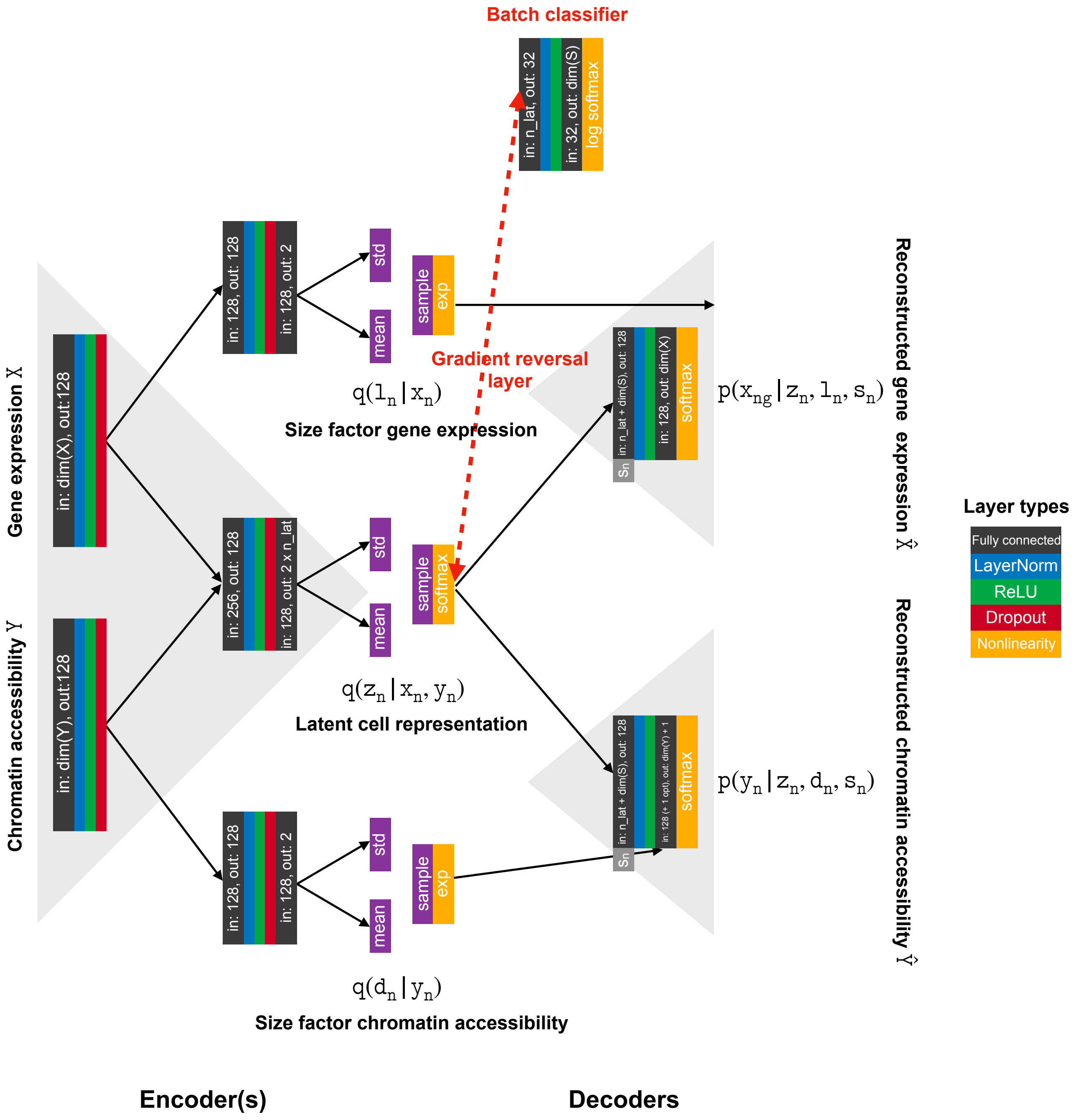

Figure S1: Liam's architecture. Schematic representation of liam's architecture for Multiome data (paired gene expression and chromatin accessibility), excluding inferred parameters. The latent cell representation z_n corresponds to the integrated embedding (default dimensionality: $k = 20$) we obtain after training. To remove batch effects, we model and infer data type- and batch-specific size factors (l_n, d_n) and dispersion parameters (not shown), and combine a conditional decoder, where we feed the one-hot encoded batch label of each cell n (s_n) to the decoder with an adversarial training strategy. The model components required for the adversarial training strategy are labeled in red. The gradient reversal layer is represented by a dashed red line. When combining paired and unimodal data (mosaic integration), we set terms with no correspondence in the loss function for cells with only a single modality measured during model training to 0. Representation inspired by Supplementary Figure 13 from [7].

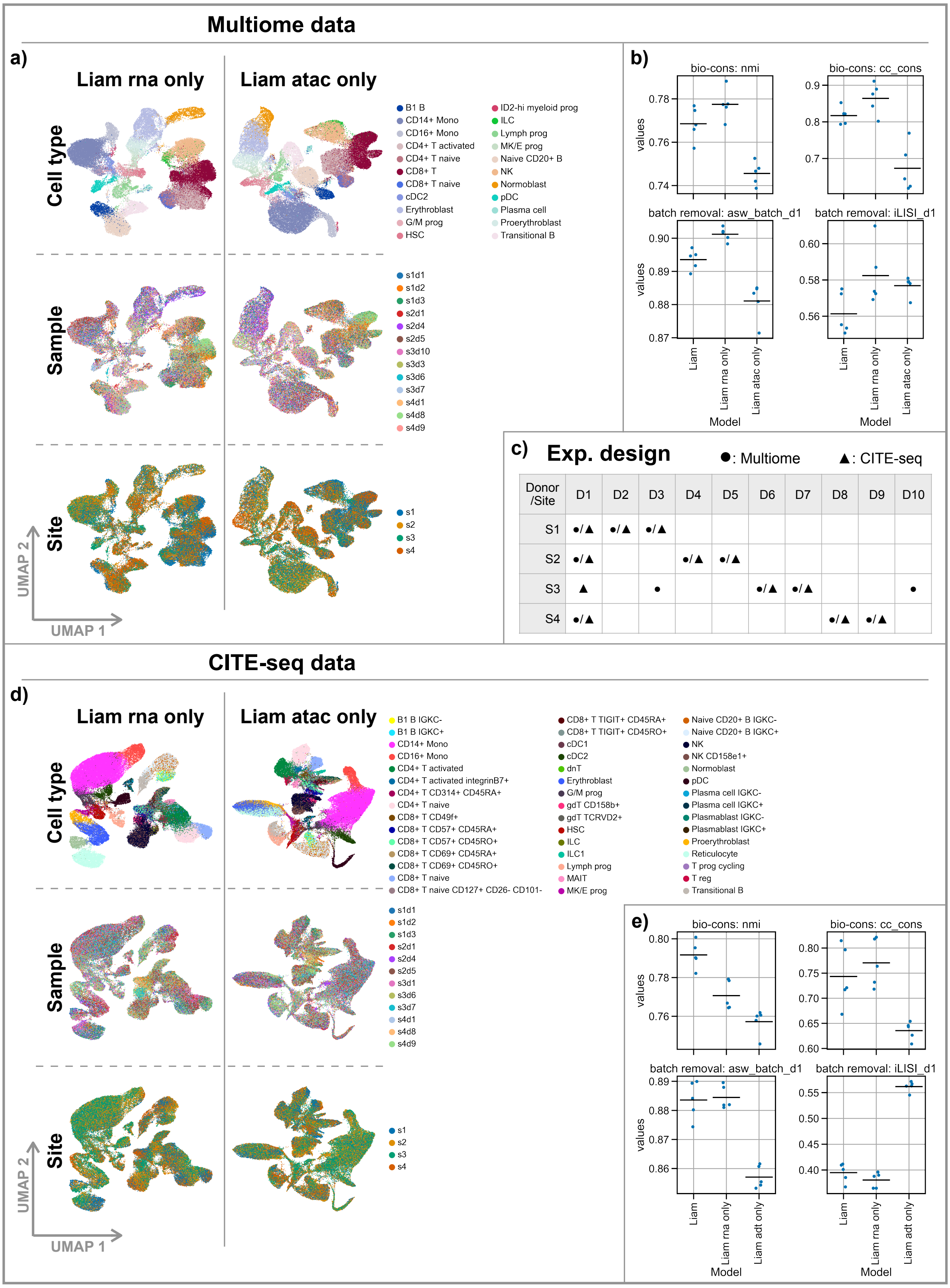

Figure S2: Performance of variants of liam using only single modalities of the multimodal data from the NeurIPS competition. Data for all figure panels stems from the NeurIPS 2021 Multimodal Single-Cell Data Integration competition. (c) Experimental design. (a) and (d) UMAPs of embeddings obtained with variants of liam using the individual modalities of (a) Multiome and (d) CITE-seq data, respectively; Cells are colored by provided cell type annotation (cell type), sample id (sample), and sequencing site (site). (b) and (e) Selected performance metrics (bio-conservation: nmi, cc_cons, batch effect removal: asw_batch_d1, iLISI_d1) with the horizontal line indicating the mean for (b) Multiome and (e) CITE-seq data. All computed metrics, including all competition metrics, are shown in Figures S7 and S9.

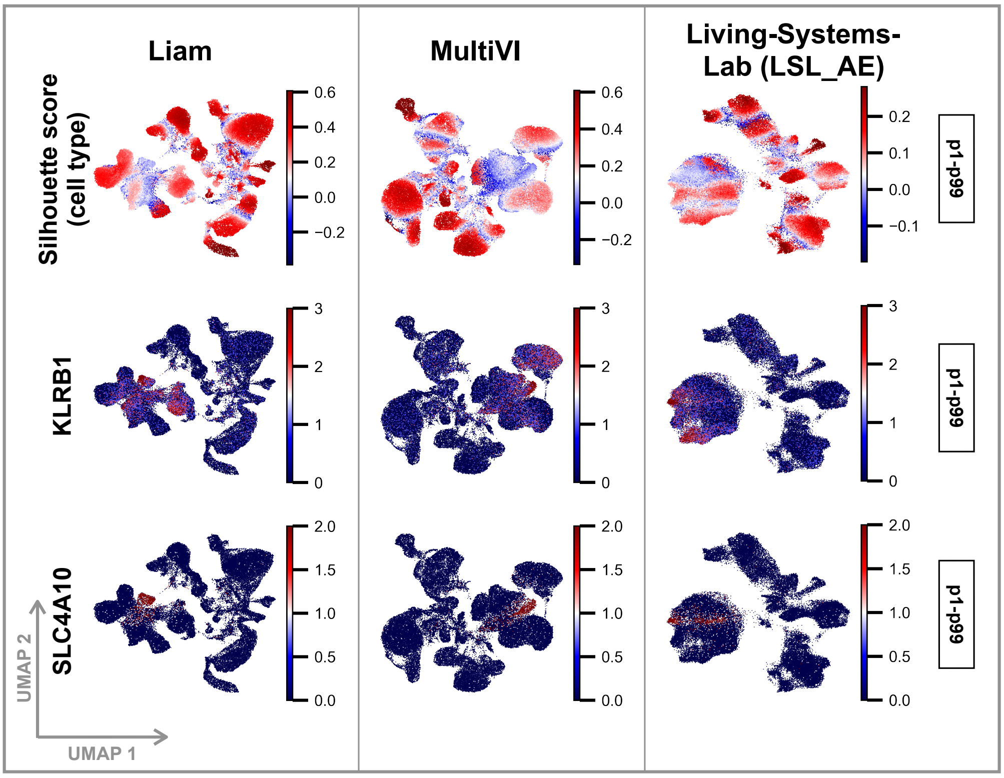

Figure S3: Liam captures cellular subpopulations missed by the NeurIPS competition reference annotation. Data for all figure panels stems from the NeurIPS 2021 Multimodal Single-Cell Data Integration competition Multiome data set. Shown are UMAP representations of the embeddings obtained with liam, MultiVI and LSL_AE with cells colored by - first row: per cell silhouette scores with respect to provided cell type annotations; second and third row: raw gene expression values for the MAIT cell markers KLRB1 and SLC4A10 [8], values outside the p1-p99 percentile range get assigned the min/max value, respectively.

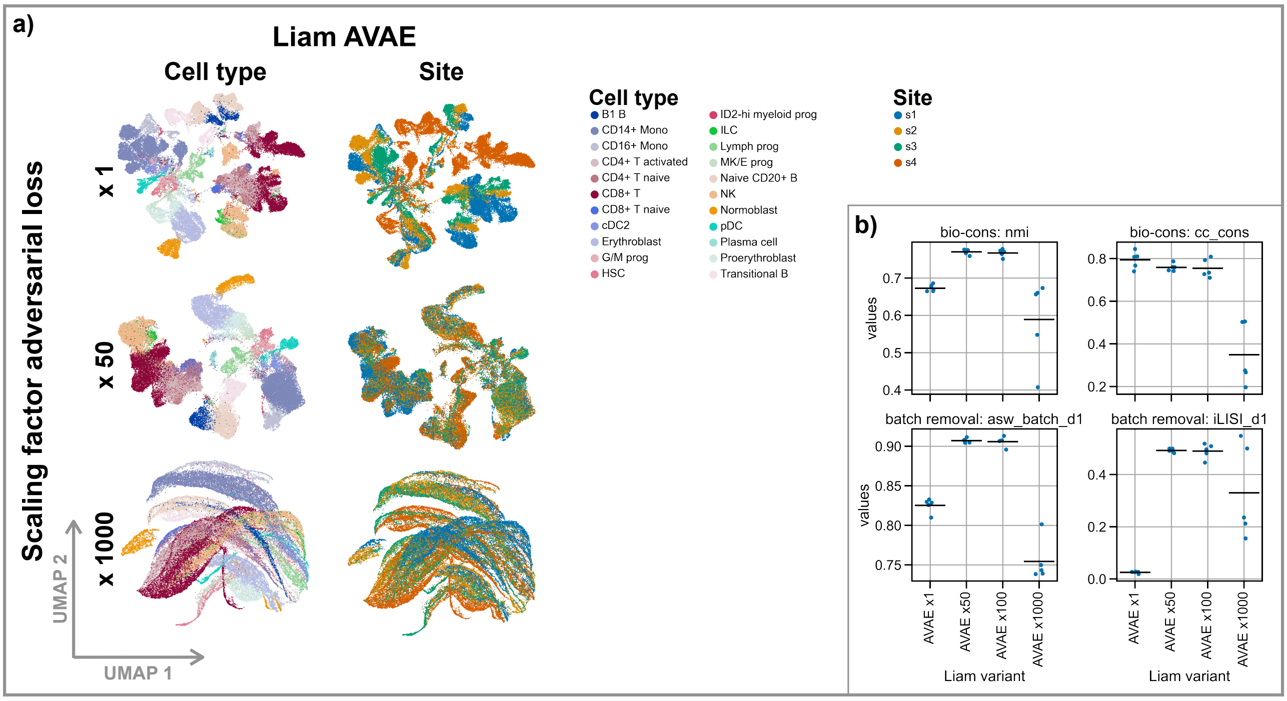

Figure S4: Adversarial training evaluation. Data for all figure panels stems from the NeurIPS 2021 Multimodal Single-Cell Data Integration competition Multiome data set. (a) UMAPs of embeddings obtained with liam AVAE with distinct contributions of the adversarial term to the overall loss function ($\alpha \in \{1, 50, 1000\}$); cells are colored by provided cell type annotations and the sequencing site (site). (b) Selected performance metrics (bio-conservation: nmi, cc_cons, batch effect removal: asw_batch_d1, iLISI_d1) with the horizontal line indicating the mean. All computed metrics, including all competition metrics, are shown in Figure S7.

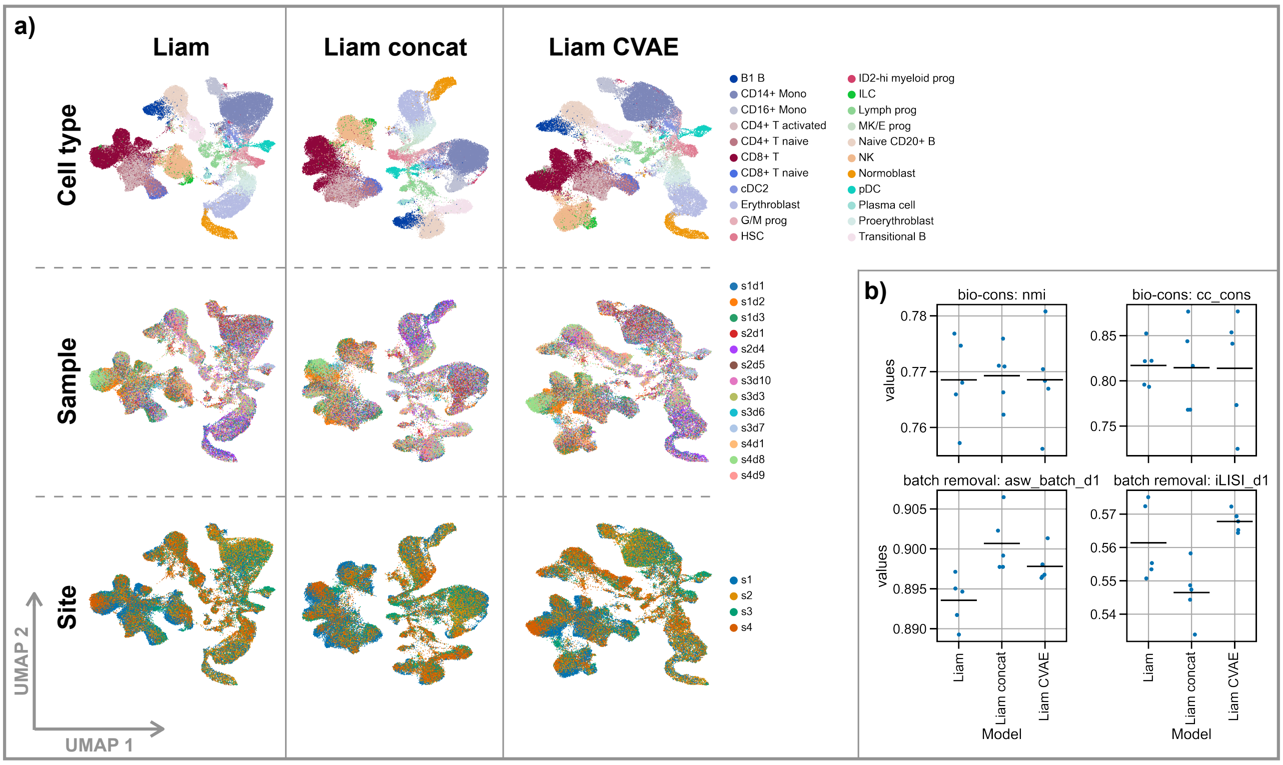

Figure S5: Liam and its baseline variants perform comparably on the competition Multiome data. Data for all figure panels stems from the NeurIPS 2021 Multimodal Single-Cell Data Integration competition Multiome data set. (a) UMAPs of embeddings obtained with distinct variants of liam; Cells are colored by provided cell type annotation (cell type), sample id (sample), and sequencing site (site). (b) Selected performance metrics (bio-conservation: nmi, cc_cons, batch effect removal: asw_batch_d1, iLISI_d1) with the horizontal line indicating the mean across models. All computed metrics, including all competition metrics, are shown in Figure S7.

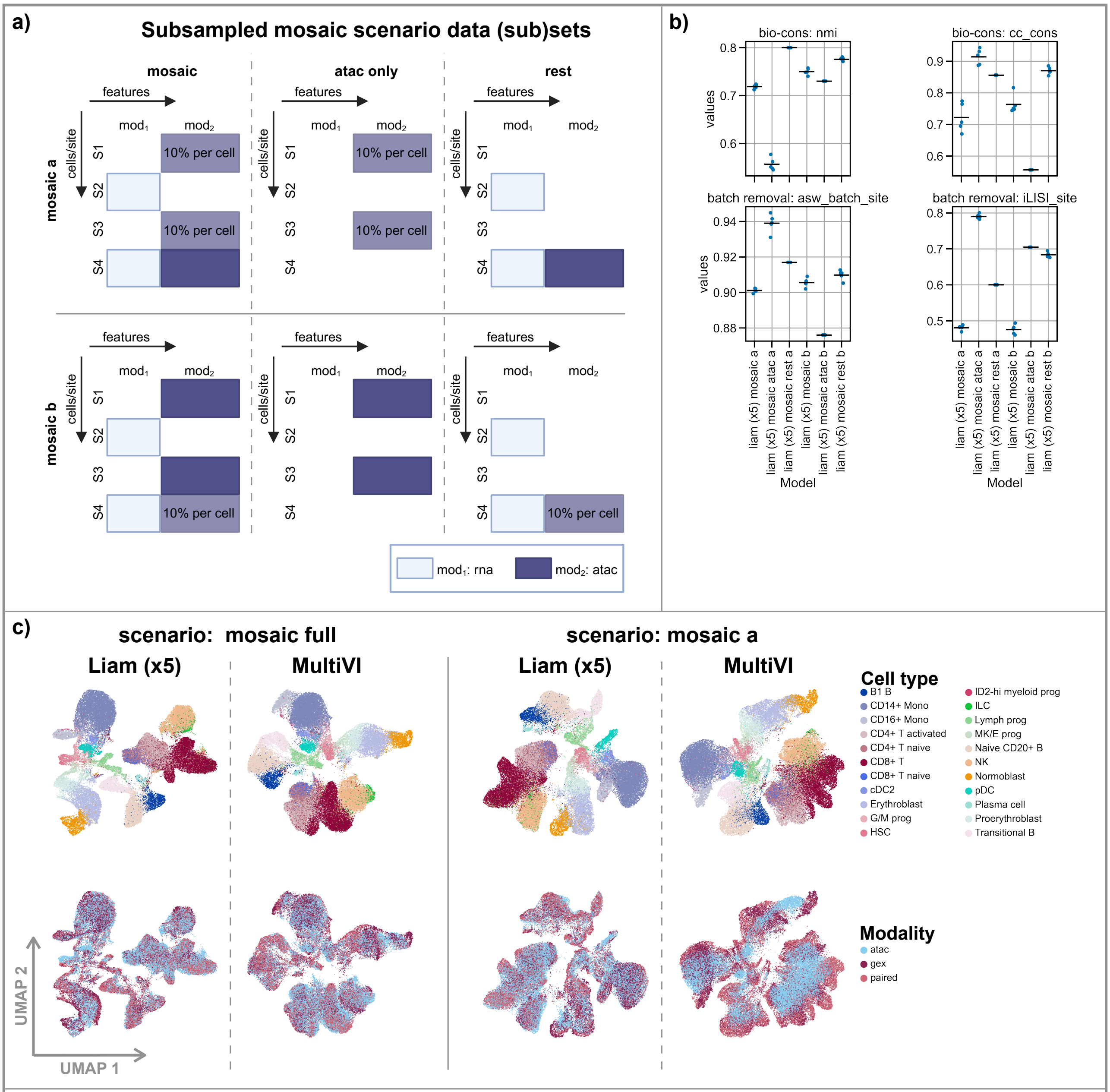

d)

Adversarial loss scaling: scenario: mosaic full

Bio-conservation

	nmi	asw_label	cc_cons	ti_cons	mean
liam (x1) mosaic full	1.8	1.8	2.4	4.0	2.5
liam (x5) mosaic full	2.4	2.0	2.4	2.0	2.2
liam (x10) mosaic full	2.4	2.2	2.4	1.6	2.1
liam (x25) mosaic full	4.0	4.0	3.0	3.0	3.5
MultiVI mosaic full	4.4	5.0	4.8	4.4	4.7

Batch removal

	asw_batch			iLISI				
	site	d1	modality	sample	site	d1	modality	mean
liam (x1) mosaic full	5.0	4.8	5.0	5.0	5.0	4.2	5.0	4.9
liam (x5) mosaic full	3.2	2.6	2.8	1.4	1.8	1.2	2.0	2.1
liam (x10) mosaic full	3.2	3.4	3.0	3.0	3.0	2.2	3.2	3.0
liam (x25) mosaic full	2.2	3.0	2.6	3.8	3.8	3.8	3.8	3.3
MultiVI mosaic full	1.4	1.2	1.6	1.8	1.4	3.6	1.0	1.7

e)

Adversarial loss scaling: scenario: mosaic a

Bio-conservation

	nmi	asw_label	cc_cons	ti_cons	mean
liam (x1) mosaic a	1.2	1.8	2.4	2.2	1.9
liam (x5) mosaic a	2.0	1.4	3.4	3.0	2.5
liam (x10) mosaic a	2.8	2.8	3.0	1.8	2.6
liam (x25) mosaic a	4.8	4.0	3.6	3.6	4.0
MultiVI mosaic a	4.2	5.0	2.6	4.4	4.0

Batch removal

	asw_batch			iLISI				
	site	d1	modality	sample	site	d1	modality	mean
liam (x1) mosaic a	2.6	2.6	2.6	3.8	3.8	3.6	3.8	3.3
liam (x5) mosaic a	3.6	2.0	1.8	1.6	2.2	1.8	2.2	2.2
liam (x10) mosaic a	2.8	1.8	1.6	1.4	1.2	1.6	1.2	1.7
liam (x25) mosaic a	5.0	3.6	4.0	3.2	2.8	3.0	2.8	3.5
MultiVI mosaic a	1.0	5.0	5.0	5.0	5.0	5.0	5.0	4.4

Figure S6: Liam excels at mosaic integration of data with differing quality (continued). Caption on next page.

Figure S6: We derived data for all figure panels from the NeurIPS 2021 Multimodal Single-Cell Data Integration competition Multiome data set (cf. Materials and methods and Figure 6(a)). (a) Data (sub)sets of the mosaic scenarios used in our evaluations (cf. (b)). If not stated otherwise, 'mosaic' is the default. For the 'mosaic full' scenario, we only consider the 'mosaic' subset. (b) Selected performance metrics (bio-conservation: nmi, cc_cons, batch effect removal: asw_batch_site, iLISI_site) with the horizontal line indicating the mean across models. (c) UMAPs of embeddings obtained with liam (α: 5) and MultiVI for the scenarios 'mosaic a' and 'mosaic full'; cells are colored by provided cell type annotations and modality. (d) and (e) Rank-based performance evaluation of model variants with varying adversarial scaling parameter using bio-conservation and batch removal metrics. Each entry corresponds to the mean rank of the model for the respective metric across five random seeds. All computed metrics, including all competition metrics, are shown in Figure S8.

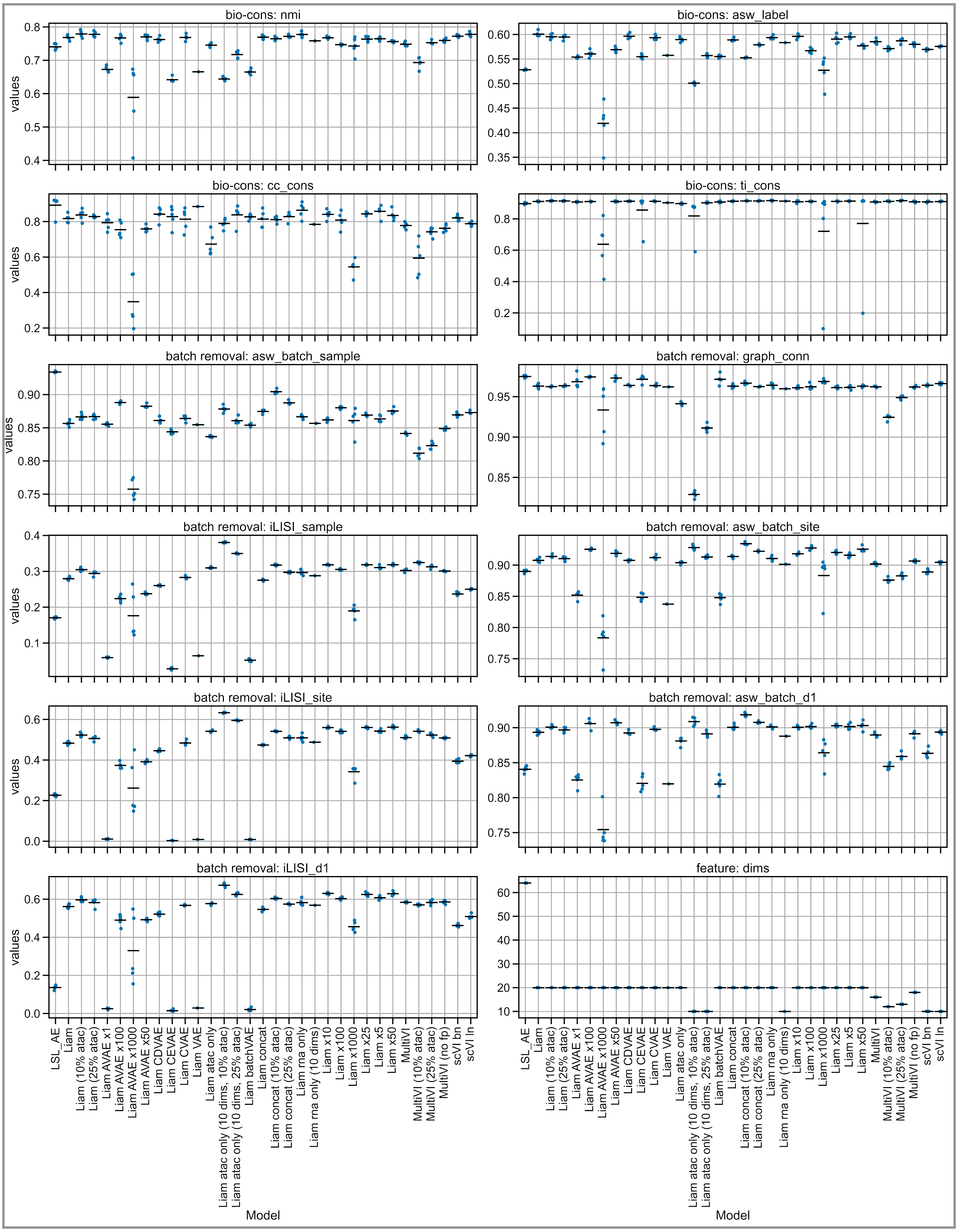

Figure S7: All evaluation metrics for all presented models and the NeurIPS 2021 Multimodal Single-Cell Data Integration competition Multiome data set (paired multimodal data integration). Shown are the results of five training runs per model, except for Liam rna only (10 dims) and Liam VAE, for which the result of a single run are shown (see Materials and methods). The horizontal line indicates the mean.

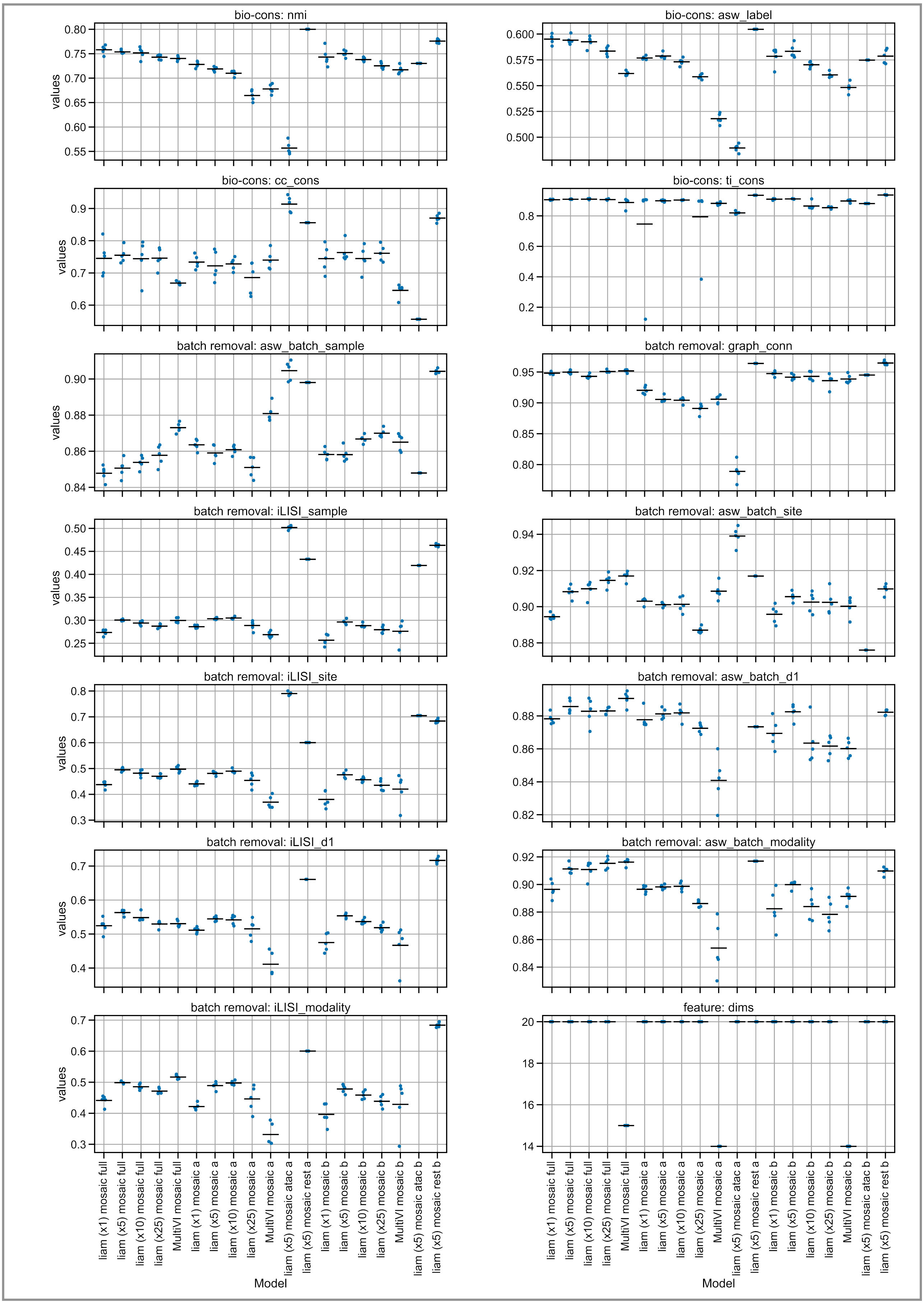

Figure S8: All evaluation metrics for all presented models trained on the mosaic use cases derived from the NeurIPS 2021 Multimodal Single-Cell Data Integration competition Multiome data set. Shown are the results of five training runs per model (see Materials and methods). The horizontal line indicates the mean.

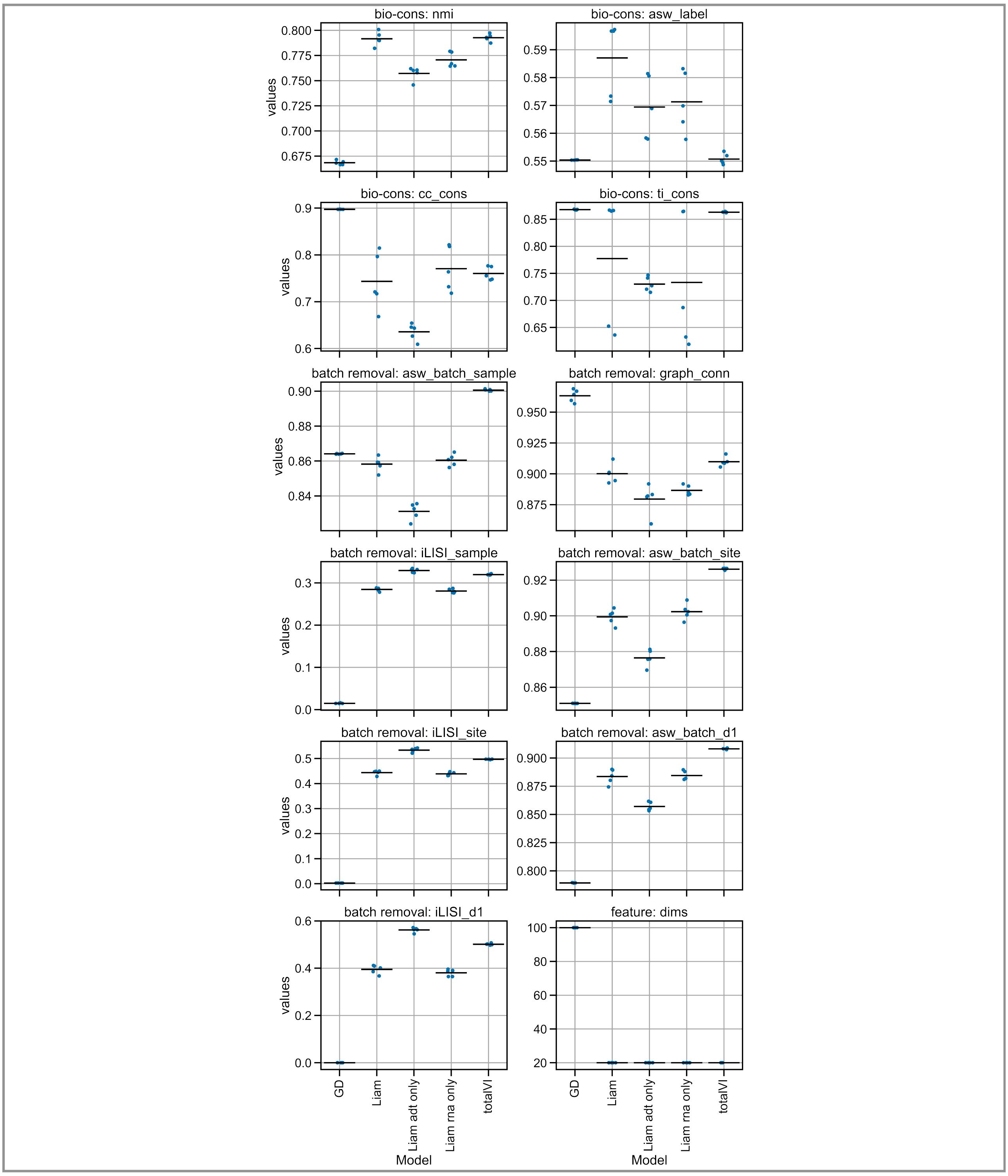

Figure S9: All evaluation metrics for all presented models on the NeurIPS 2021 Multimodal Single-Cell Data Integration competition CITE-seq data set (paired multimodal data integration). Shown are the results of five training runs per model (see Materials and methods); the horizontal line indicates the mean. GD: Guanlab-dengkw.
